# Multivariate study of lice (Insecta: Psocodea: Phthiraptera) assemblages hosted by hummingbirds (Aves: Trochilidae)

**DOI:** 10.1017/S0031182023001294

**Published:** 2024-02

**Authors:** Oldřich Sychra, Lajos Rózsa, János Podani, Vojtěch Sychra, Ivan Literák, Miroslav Capek

**Affiliations:** 1 Department of Biology and Wildlife Diseases, Faculty of Veterinary Hygiene and Ecology, University of Veterinary Sciences, Brno, Czechia; 2 Institute of Evolution, Centre for Ecological Research, Budapest, Hungary; 3 Centre for Eco-Epidemiology, National Laboratory for Health Security, Budapest, Hungary; 4 Hungarian Department of Biology and Ecology, Babeș-Bolyai University, Cluj-Napoca, Romania; 5 Department of Plant Systematics, Ecology and Theoretical Biology, Institute of Biology, Eötvös University, Budapest, Hungary; 6 Institute of Vertebrate Biology, Czech Academy of Sciences, Brno, Czechia

**Keywords:** Abundance, d-correlation, elevation, migration, multivariate study, ordination, prevalence, sexual dimorphism, *Trochiliphagus*, *Trochiloecetes*

## Abstract

Lice were collected from 579 hummingbirds, representing 49 species, in 19 locations in Brazil, Costa Rica, Honduras, Paraguay and Peru, at elevations 0–3000 m above sea level. The following variables were included in an ecological analysis (1) host species' mean body mass, sexual size dimorphism, sexual dichromatism, migratory behaviour and dominance behaviour; (2) mean elevation, mean and predictability of temperature, mean and predictability of precipitation of the host species' geographic area; (3) prevalence and mean abundance of species of lice as measures of infestation. Ordination methods were applied to evaluate data structure. Since the traits are expressed at different scales (nominal, interval and ratio), a principal component analysis based on d-correlations for the traits and a principal coordinates analysis based on the Gower index for species were applied. Lice or louse eggs were found on 80 (13.8%) birds of 22 species. A total of 267 lice of 4 genera, *Trochiloecetes*, *Trochiliphagus*, *Myrsidea* and *Leremenopon*, were collected, with a total mean intensity of 4.6. There were positive interactions between migration behaviour and infestation indices, with elevational migrants having a higher prevalence and abundance of lice than resident birds. Further, we found weak negative correlations between host body mass and infestation indices and positive correlations between mean elevation and prevalence and abundance of *Trochiliphagus*. Thus, formerly unknown differences in the ecological characteristics and infestation measures of *Trochiliphagus* and *Trochiloecetes* lice were revealed, which allows a better understanding of these associations and their potential impacts on hummingbirds.

## Graphical abstract

**Figure:**
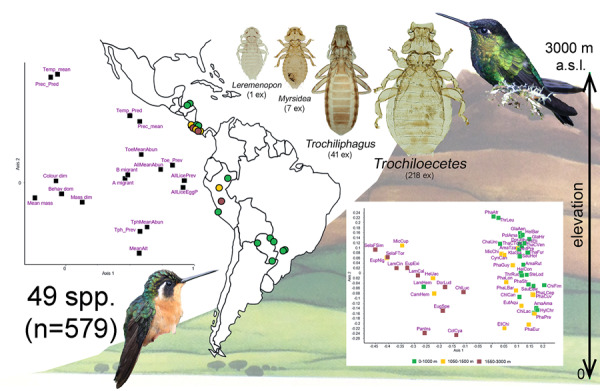

## Introduction

Parasitic lice (Psocodea: Phthiraptera) are among the most common members of the avian ectoparasite fauna. They complete their whole life cycle on the skin surface and in the plumage of birds, and they are primarily transmitted through direct body-to-body contact between birds (Johnson and Clayton, 2003). They constitute ideal model organisms to study the ecology of contagious pathogens simply because they are relatively large, easily found, observed, collected and counted even by the naked eye.

In comparisons across species, the prevalence (proportion of infested individuals) and mean abundance (mean number of parasites per host) of lice typically covary positively with host body size (Rothschild and Clay, 1952; Rózsa, 1997). This prompts the question of which host traits, and which environmental variables affect lice assemblages of hummingbirds – the smallest birds on Earth.

Three hundred and sixty-six species of hummingbirds (Trochilidae) have been recognized (Gill *et al.*, 2023). Despite this relatively large number of host species, only 50 species of 4 genera of lice – *Leremenopon*, *Myrsidea*, *Trochiloecetes* and *Trochiliphagus* – have been described so far from only 37 (10%) species of hummingbirds (Dalgleish and Price, 2003*a*, 2003*b*; Price *et al.*, 2003). Recently, Oniki-Willis *et al.* (2023) documented the presence of louse eggs on 291 (80%) species, indicating that current knowledge about louse communities associated with hummingbirds is still scarce and incomplete.

*Leremenopon* and *Myrsidea* belong to the family Menoponidae. Menoponids are widespread on many other avian taxa, but scarce on hummingbirds, having extremely low prevalences (<1%) with mostly 1 louse per bird (Dalgleish and Price, 2003*a*, 2003*b*; Oniki-Willis *et al.*, 2023).

On the other hand, members of the family Ricinidae are specialized to small-bodied hosts, namely small-bodied (or, less frequently, medium-sized) passerines and hummingbirds (Harnos *et al.*, 2016). However, if related to host size, they are large bodied themselves. Two genera occur on hummingbirds, *Trochiliphagus* and *Trochiloecetes*, which are more frequent (prevalence often between 5 and 15%) than menoponids. They consume host blood, an exceptional feeding strategy for avian lice (Clay, 1949; Carriker, 1960). Oniki-Willis *et al.* (2023) documented that the co-occurrence of 2 genera on the same host individual was more frequent than expected by chance. However, the reasons for this positive covariation are unknown, because this analysis was based on eggshells collected from museum specimens. No further information is available on the parasites' impact on hummingbirds or the environmental factors that may affect their distribution and abundance.

Almost nothing is known about the host-specificity of ricinid lice infesting hummingbirds. While Price *et al.* (2003) and former authors treated species of both genera as strictly host-specific, Rheinwald (2007) suggested that all 13 known species of *Trochiliphagus* are, in fact, the same species. Moreover, he suggested that this species belongs to the genus *Ricinus* – a genus including only host-generalist lice of Passerines (Price *et al.*, 2003). This debate is rooted in the uncertainty of the species concept in parasitology (Mey, 1998; Gustafsson and Najer, 2022). Rheinwald (2007) based his study on investigation of only 2 *Trochiliphagus* specimens from the same host, and did not examine any of the type material. Therefore, until proper morphometric revision of type material of *Trochiliphagus* species and genetic analysis of specimens from different hummingbirds are provided, we consider Rheinwald's hypothesis insufficiently supported (see also Valan *et al.,* 2016).

The purpose of the present study is to explore host traits and environmental variables potentially affecting the louse communities of hummingbirds in Central and South America.

## Materials and methods

Lice were collected from hummingbirds mist netted from July to September 2004–2014 in 19 locations in Costa Rica, Brazil, Honduras, Paraguay and Peru (Additional file 1: Table S1). These collection sites were situated in primary rainforests, pastures or gardens near forests at elevations from 0 to 3000 m above sea level (a.s.l.) (Additional file 1: Table S1). Mist nets were inspected every 30–40 minutes, and captured hummingbirds were placed into separate cloth bags. Hummingbird identification was based on illustrated identification field guides (Stiles and Skutch, 1989; Howell and Webb, 1995; Narosky and Yzurieta, 2006, 2010; Sigrist, 2006; Garrigues, 2007; Gwynne *et al.*, 2010; Schulenberg *et al.*, 2010), while their taxonomy and nomenclature follow Gill *et al.* (2023).

The fumigation chamber method was applied to collect lice from the birds, using chloroform as a fumigant for 5–7 min (Clayton and Drown, 2001). This was complemented by a visual search of the head for the occurrence of louse eggs. Contrary to Oniki-Willis *et al.* (2023), eggs or nits (the empty chorions of hatched eggs) were not identified to the genus level. Birds were released immediately after examination. Lice were stored in 96% ethanol and slide-mounted in Canada balsam as permanent slides.

Host species were characterized by their mean body mass (male and female masses averaged), based on del Hoyo *et al.* (1999). Male body mass was regressed over female body mass, and the residuals from this regression line were used as the index of sexual size dimorphism (SSD). The body mass and SSD values of *Stephanoxis loddigesii* were apparently affected by a typographical error and, therefore, excluded from the analysis. Binary categorization for sexual dichromatism (different colouration of males and females) was adopted from Diamant *et al.* (2021). In the case of 4 species (*Amazilia rutila*, *Amazilia tzacatl*, *Heliomaster constantii* and *Saucerottia hoffmanni*), dichromatism categorization was modified based on the authors' field experiences and the literature (see list of publications in Additional file 1: Text S1). To quantify potentially relevant aspects of host behaviour, 2 alternative indices to categorize migration (both obtained from del Hoyo *et al.*, 1999) were included. To describe the behaviourally dominant nature (aggressive against rival species or not) of hummingbird species, the binary categorization of Bribiesca *et al.* (2019) was applied.

Geographic area for each species (and not the actual collection sites) was characterized by mean elevation (a.s.l.), mean and predictability of temperature, and mean and predictability of precipitation. All these data were obtained from Diamant *et al.* (2021). In the following 4 cases, names of different taxa were applied:

- *Eugenes spectabilis* was formerly treated as a subspecies, i.e., *E. fulgens spectabilis*. Therefore, data of *E. fulgens* were used for this taxon;
- *Saucerottia hoffmanni* was formerly treated as a subspecies, i.e., *S. saucerottei hoffmanni*. Therefore, data of *Saucerottia saucerottei* were used for this taxon;
- To characterize *Phaethornis striigularis*, data were obtained from del Hoyo *et al.* (1999), except for temperature and precipitation, which were assessed by adopting the data of *Phaethornis longirostris*, a species having a similar area of distribution (del Hoyo *et al.*, 1999);
- To characterize *Colibri cyanotus*, data were obtained from del Hoyo *et al.* (1999), except for temperature and precipitation, which were taken from *Colibri delphinae*, a species inhabiting a similar distribution area (del Hoyo *et al.*, 1999).

Other relevant literature (Additional file 1: Text S1) was also used to confirm these decisions.

Prevalence (the proportion of infested individuals) and mean abundance (mean number of lice per host individual, including 0 values of non-infested birds) were calculated separately for each genus of parasite found infesting all hummingbird species. Further, treating all taxa of lice as members of the same ecological guild, the prevalence and mean abundance were calculated pooled for all the lice together. There were 2 different ways to quantify the prevalence of all lice. First, only present infestations were considered (all birds that harboured adults and/or nymphs). Then, past infestations were also added to the infested category by including those birds which harboured only eggs or nits in their plumage. This latter method includes present and former infestations that might have already disappeared at the time of collection. This might also include birds on which hatched lice were undetected by the collection techniques used. This latter index of prevalence (with eggs and nits considered) was calculated only when data for all lice were pooled into a guild, but not for louse genera taken separately (because eggs were not identified to genus level). Fisher's exact test was applied to compare prevalences and bootstrap 2-sample *t*-test was used to compare mean intensities (the number of lice per infested host). Calculations were carried out using Quantitative Parasitology 3.0 (Rózsa *et al.*, 2000).

Since infestation indices are not specific to hosts or parasites but reflect relationships for host–parasite species pairs, phylogenetic control for comparative analyses was not applied. Instead, ordination methods were used to explore the correlation structure among variables and to evaluate the distances between species based on these variables. They were measured at different scales (nominal, interval and ratio). Therefore, the d-correlation approach (Podani *et al.*, 2023) was used to calculate correlations and subsequent principal component analysis of variables, and Gower's dissimilarity (Gower, 1971) and subsequent principal coordinates analysis of species and subspecies. Computations were made by programs DCORR and SYN-TAX 2000 (available from http://podani.web.elte.hu/SYN2000.html).

## Results

A total of 579 individual hummingbirds were examined. They represented 49 species, of which 3 were divided into 2–3 subspecies, resulting in a total of 53 separate host taxa to be included in the multivariate analysis. Only 59 (10.2%) individual birds and 20 (40.8%) species of birds were parasitized when considering only adult and nymphal stages of lice. When hosts with louse eggs and nits (indicating past infestations) were also included in the parasitized category, 80 (13.8%) birds of 22 (44.9%) species were infested.

Neither louse nor louse eggs or nits were found on 27 species of hummingbirds. As expected, the number of host individuals correlated positively with the number of louse species (Spearman rank correlation coefficient *ρ* = 0.52, *P* = 0.0001). Thus, small sample size probably accounted for the absence of lice on certain host taxa. Small sample sizes significantly increase randomness in prevalence and abundance data; however, they do not systematically bias these estimates (Reiczigel and Rózsa, 2017). Therefore, small samples were not excluded from the multivariate analysis. A total of 267 lice representing 4 genera were collected, with a total mean intensity of 4.6 and mean abundance of 0.5. For further details, see Table 1 and Additional file 1: Tables S2–S4. For the same reason, data regarding the age of the hummingbirds examined were not included (Additional file 1: Table S5).

**Table 1.** Infestation indices of lice collected from hummingbirds in the present study

Country	Examined birds (number of species)	Infested birds (adults or nymphs) (number of species)	Infested birds (adults or nymphs or eggs) (number of species)	Number of lice (adults or nymphs)	Prevalence (adults or nymphs) (%)	Prevalence (adults or nymphs or eggs) (%)	Mean intensity (adults or nymphs)	Mean abundance (adults or nymphs)
Brazil	4 (2)	0	0	0	0	0	0	0
Costa Rica	428 (32)	42 (15)	56 (16)	164	9.8	13.1	4.0	0.4
Honduras	87 (9)	11 (3)	15 (5)	54	12.6	17.2	5.0	0.6
Paraguay	13 (4)	0	0	0	0	0	0.0	0
Peru	47 (10)	6 (4)	9 (4)	49	12.8	19.1	8.2	1.0
Total	579 (49)	59 (20)	80 (22)	267	10.2	13.8	4.6	0.5

The proportion of individuals belonging to each genus, *Trochiloecetes* 81.6%, *Trochiliphagus* 15.4%, *Myrsidea* 2.6% and *Leremenopon* 0.4% (*n* = 267).

A total of 29 louse associations for 20 species of hummingbird were documented and evaluated (Table 2). On 13 species, only 1 genus of lice was recorded. On 5 species, *Trochiloecetes* and *Trochiliphagus*; on 1 species, *Trochiloecetes* and *Myrsidea*; on 1 species, *Trochiloecetes* and *Leremenopon* were recorded. The co-occurrence of *Trochiloecetes* and *Trochiliphagus* was detected only on 1 individual of *Selasphorus flammula* from Costa Rica (Table 2). *Myrsidea* and *Leremenopon* were excluded from all further analyses due to their rarity.

**Table 2.** List of hummingbirds and lice recorded from them by the authors in Costa Rica, Honduras and Peru. *P*, number of birds parasitized; *E*, number of birds examined. For explanation of abbreviations of locations, see Additional file 1: Table S1

Host group and (sub)species	*P*	*E*	Louse genus	Females	Males	Nymphs	Location	Elevation
Hermits (Phaethornithinae)								
*Phaethornis atrimentalis atrimentalis* Lawrence, 1858^a^	2	4	*Trochiloecetes*^b^	2			Pe_TR	779
*Phaethornis longirostris baroni* Hartert, EJO, 1897	1	5	*Trochiloecetes*^b^	4	5	4	Pe_TR	779
<< ″ ″ ″ >>	0^+1^^c^	5					Pe_IQ	128
*Phaethornis longirostris cephalus* (Bourcier and Mulsant, 1848)	0	27					CR_HC	141
<< ″ ″ ″ >>	0	11					CR_B	520
*Phaethornis longirostris longirostris* (Delattre, 1843)	1	27	*Myrsidea*^b^		1	6	Ho_LA	256
<< ″ ″ ″ >>	8	27	*Trochiloecetes*^b^	20	2	8	Ho_LA	256
*Phaethornis striigularis saturatus* Ridgway, 1910^a^	1^+1^^c^	2	*Trochiliphagus*^b^	1			CR_RV	934
<< ″ ″ ″ >>	0	9					CR_HC	141
<< ″ ″ ″ >>	0	2					CR_T	1333
<< ″ ″ ″ >>	0	2					Ho_LA	256
*Threnetes leucurus cervinicauda* Gould, 1855	1	3	*Trochiloecetes*	7	4	1	Pe_TR	779
*Threnetes ruckeri ventosus* Bangs and Penard, TE, 1924^a^	2^+1^^c^	17	*Trochiloecetes*^b^	4	0	3	CR_B	520
<< ″ ″ ″ >>	1	12	*Trochiloecetes*	0	0	2	CR_HC	141
<< ″ ″ ″ >>	1	1	*Trochiloecetes*	6	1	2	Ho_LA	256
Mangoes (Polythminae)								
*Anthracothorax prevostii gracilirostris* Ridgway, 1910	0^+1^^c^	1					Ho_LA	256
Brilliants (Lesbiinae – Coeligenini)								
*Heliodoxa jacula henryi* Lawrence, 1867^a^	1	10	*Trochiliphagus*^b^	1			CR_T	1333
Mtn. Gems (Trochilinae – Lampornini)								
*Eugenes spectabilis* (Lawrence, 1867)	2^+2^^c^	25	*Trochiliphagus*^b^	2		1	CR_CM	2987
<< ″ ″ ″ >>	1	25	*Trochiloecetes*^b^	1			CR_CM	2987
<< ″ ″ ″ >>	0	1					CR_BV	2229
*Lampornis calolaemus calolaemus* (Salvin, 1865)^a^	4^+3^^c^	28	*Trochiloecetes*^b^	6	5	2	CR_BV	2229
<< ″ ″ ″ >>	0	1					CR_T	1333
*Lampornis cinereicauda* (Lawrence, 1867)^a^	2	5	*Trochiloecetes*^b^	6	4		CR_CM	2987
*Lampornis hemileucus* (Salvin, 1865)^a^	1	8	*Trochiliphagus*^b^			1	CR_T	1333
*Panterpe insignis insignis* Cabanis and Heine, 1860^a^	4^+1^^c^	41	*Trochiloecetes*^b^	14	9	2	CR_CM	2987
<< ″ ″ ″ >>	0	1					CR_BV	2229
Bees (Trochilinae – Mellisugini)								
*Selasphorus flammula simoni* Carriker, 1910	2^+1c, d^	13	*Trochiliphagus*	6	4	3	CR_BV	2229
<< ″ ″ ″ >>	6^d^	13	*Trochiloecetes*	6		4	CR_BV	2229
*Selasphorus flammula torridus* Salvin, 1870	1^+1^^c^	10	*Trochiliphagus*		2	3	CR_CM	2987
<< ″ ″ ″ >>	3	10	*Trochiloecetes*	1	1	3	CR_CM	2987
Emeralds (Trochilinae – Cynanthini)								
*Amazilia tzacatl tzacatl* (de la Llave, 1833)	0^+2^^c^	33					Ho_LA	256
<< ″ ″ ″ >>	0	4					CR_B	520
<< ″ ″ ″ >>	0^+1^^c^	9					CR_HC	141
<< ″ ″ ″ >>	0^+1^^c^	4					CR_LT	1413
*Campylopterus hemileucurus mellitus* Bangs, 1902	1	35	*Trochiloecetes*^b^	14	7	12	CR_T	1333
<< ″ ″ ″ >>	0	5					CR_LT	1413
<< ″ ″ ″ >>	0	3					CR_BV	2229
*Cynanthus canivetii salvini* (Cabanis and Heine, 1860)^a^	1	3	*Trochiliphagus*^b^			1	CR_RV	934
<< ″ ″ ″ >>	0	4					Ho_UT	4
*Elliotomyia chionogaster chionogaster* (Tschudi, 1846)^a^	1^+2^^c^	19	*Trochiliphagus*^b^	2			Pe_HU	2365
<< ″ ″ ″ >>	1	19	*Trochiloecetes*^b^	5	9	6	Pe_HU	2365
*Eupherusa eximia egregia* Sclater, PL and Salvin, 1868^a^	1^+1^^c^	6	*Trochiliphagus*^b^	1			CR_RV	934
<< ″ ″ ″ >>	1	6	*Trochiloecetes*^b^		2	3	CR_RV	934
<< ″ ″ ″ >>	1	1	*Trochiloecetes*	1		1	CR_T	1333
<< ″ ″ ″ >>	0^+1^^c^	1					CR_BV	2229
*Eupherusa nigriventris* Lawrence, 1868^a^	2	5	*Trochiliphagus*^b^	1	1	3	CR_T	1333
<< ″ ″ ″ >>	1	5	*Trochiloecetes*^b^	1		1	CR_T	1333
*Chalybura urochrysia melanorrhoa* Salvin, 1865^a^	1	7	*Leremenopon*^b^	1	0	0	CR_B	520
<< ″ ″ ″ >>	2	13	*Trochiloecetes*^b^	3	1	12	CR_HC	141
*Chlorestes candida candida* (Bourcier and Mulsant, 1846)	1^+1^^c^	13	*Trochiliphagus*		3	5	Ho_LA	256
*Microchera cupreiceps* (Lawrence, 1866)^a^	1	1	*Trochiloecetes*^b^			1	CR_RV	934
Total	59^+21^^c^	579		116	61	90		

a first recorded louse from this host.

b new host-louse association

c only eggs of unidentified lice were found on the indicated number of other individuals.

d one bird was parasitized by lice of both genera.

While *Trochiloecetes* was more prevalent than *Trochiliphagus* (7.6% *vs* 2.4%; *P* < 0.001), there was no significant difference between their mean intensities (5.0 *vs* 2.9; *P* = 0.11). Female-biased sex ratio and adult-biased age ratio were found for *Trochiloecetes* (male:female = 1:2; *n* = 151; *χ*^2^ = 17, *P* < 0.001; adults:nymphs = 1:0.4; *n* = 218; *χ*^2^ = 32, *P* < 0.001), but ratios were more equal for *Trochiliphagus* (male:female = 1:1.4; *n* = 24; *χ*^2^ = 0.66, *P* > 0.05; adults:nymphs = 1:0.7; *n* = 41; *χ*^2^ = 1.2, *P* > 0.05) (Additional file 1: Table S6).

Most infested birds (90%, *n* = 59) were parasitized with 1–10 lice. A total of 11–20 lice were found on 5 birds (*Elliotomyia chionogaster, Chalybura urochrysia, Panterpe insignis, P. longirostris, Threnetes leucurus*) and 1 *Campylopterus hemileucurus* was parasitized with 33 lice. In all cases, these birds were infested only with *Trochiloecetes*. Intensity of infestations with *Trochiliphagus* were 1–10 per infested host.

**Figure 1.**
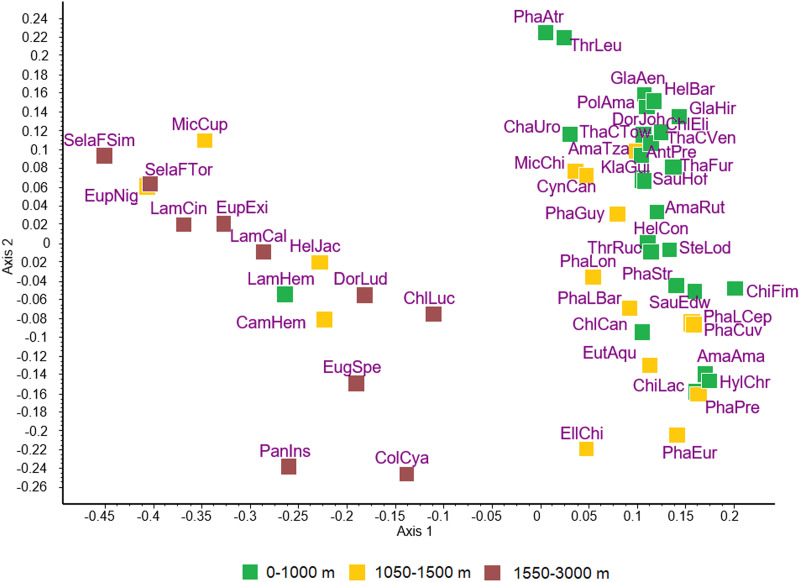
Principal coordinates ordination of 49 species of hummingbirds for the first 2 dimensions accounting for 29.5% and 11.3% of variance, respectively. Subspecies are recognized for *Phaethornis longirostris*, *Selasphorus flammula* and *Thalurania colombica*, resulting in a total of 53 host taxa that were included in the analysis: AmaAma, *Amazilis amazilia*; AmaRut, *Amazilia rutila*; AmaTza, *Amazilia tzacatl*; AntPre, *Anthracothorax prevostii*; CamHem, *Campylopterus hemileucurus*; ChaUro, *Chalybura urochrysia*; ChiFim, *Chionomesa fimbriata*; ChiLac, *Chionomesa lactea*; ChlCan, *Chlorestes candida*; ChlEli, *Chlorestes eliciae*; ChlLuc, *Chlorostilbon lucidus*; ColCya, *Colibri cyanotus*; CynCan, *Cynanthus canivetii*; DorJoh, *Doryfera johannae*; DorLud, *Doryfera ludovicae*; EllChi, *Elliotomyia chionogaster*; EugSpe, *Eugenes spectabilis*; EupExi, *Eupherusa eximia*; EupNig, *Eupherusa nigriventris*; EutAqu, *Eutoxeres aquila*; GlaAen, *Glaucis aeneus*; GlaHir, *Glaucis hirsutus*; HelBar, *Heliothryx barroti*; HelCon, *Heliomaster constantii*; HelJac, *Heliodoxa jacula*; HylChr, *Hylocharis chrysura*; KlaGui, *Klais guimeti*; LamCal, *Lampornis calolaemus*; LamCin, *Lampornis cinereicauda*; LamHem, *Lampornis hemileucus*; MicCup, *Microchera cupreiceps*; MicChi, *Microchera chionura*; PanIns, *Panterpe insignis*; PhaAtr, *Phaethornis atrimentalis*; PhaCuv, *Phaeochroa cuvierii*; PhaEur, *Phaethornis eurynome*; PhaGuy, *Phaethornis guy*; PhaLBar, *Phaethornis longirostris baroni*; PhaLCep, *Phaethornis longirostris cephalus*; PhaLon, *Phaethornis longirostris longirostris*; PhaPre, *Phaethornis pretrei*; PhaStr, *Phaethornis striigularis*; PolAma, *Polyerata amabilis*; SauEdw, *Saucerottia edward*; SauHof, *Saucerottia hoffmanni*; SelaFSim, *Selasphorus flammula simoni*; SelaFTor, *Selasphorus flammula torridus*; SteLod, *Stephanoxis loddigesii*; ThaCTow, *Thalurania colombica townsendi*; ThaCVer, *Thalurania colombica venusta*; ThaFur, *Thalurania furcata*; ThrLeu, *Threnetes leucurus*; ThrRuc, *Threnetes ruckeri*.

The ordination of hummingbird species is shown in Fig. 1. Similarities or differences between species as indicated by the distances among them do not correspond to the taxonomic divisions within the Trochilidae. Rather, the 2 major groups of species appear to include species of hummingbirds found at low elevation *vs* high elevation.

**Figure 2.**
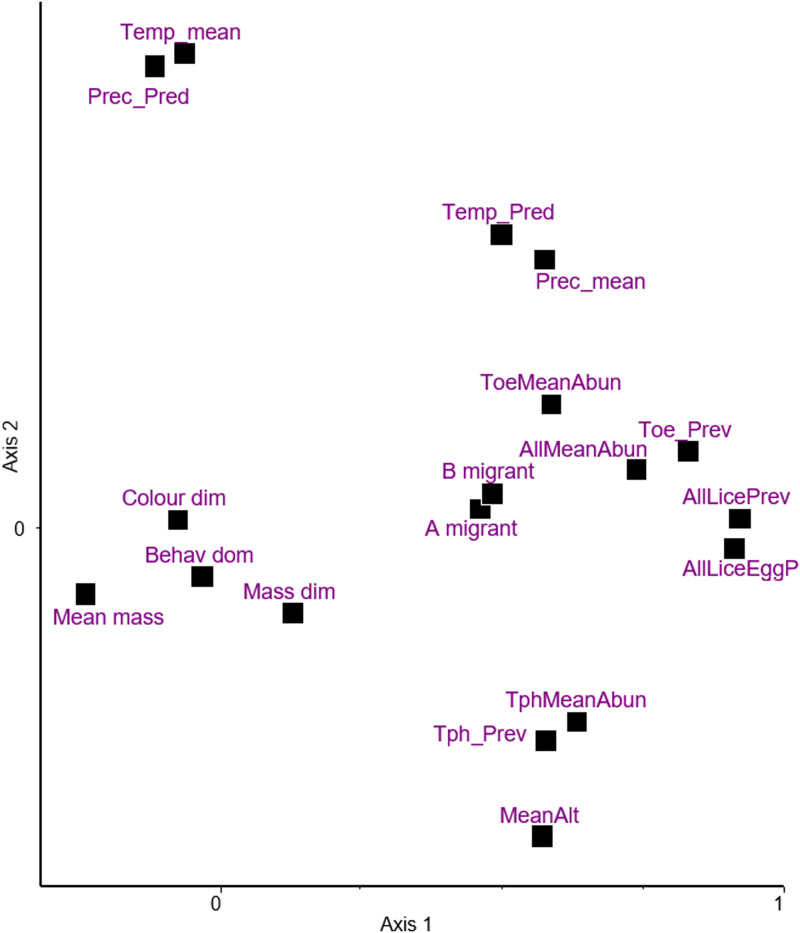
Principal component analysis of variables; climatic and host traits, and infestation indices on the first 2 dimensions explaining 30% and 15% of variance, respectively. Following variables are included: (A) hummingbirds' characteristics: A migrant – migrant behaviour, B migrant – type of migration, Behav_dom, behavioural dominance; Colour_dim, sexual dichromatism; Mass_dim, sexual size dimorphism; Mean_mass, mean body mass; (B) characteristics of geographic areas: MeanAlt, mean elevation; Prec_mean, mean precipitation; Prec_Pred, predictability of precipitation; Temp_mean, mean temperature; Temp_Pred, predictability of temperature; (C) infestation indices: AllLiceEggP, prevalence of lice and louse eggs or nits; AllLicePrev, prevalence of lice; AllMeanAbun, mean abundance of lice; ToeMeanAbun, mean abundance of *Trochiloecetes* lice; Toe_Prev, prevalence of *Trochiloecetes* lice; TphMeanAbun, mean abundance of *Trochiliphagus* lice; Tph_Prev, prevalence of *Trochiliphagus* lice.

Principal components ordination of climatic and host traits together with the infestation indices is shown in Fig. 2, the first 2 dimensions explaining 30 and 15% of variance, respectively. Based on this and the matrix of d-correlations (Additional file 1: Table S8) the following points can be observed:

- Sexual dimorphism in colouration and body mass, and behavioural dominance over rival species are close to the origin, indicating little, if any, covariation with other variables. The highest correlation for mass dimorphism is with mean elevation (*r* = 0.222), whereas all correlations for colouration dimorphism remain *r* ≤ 0.074. Behavioural dominance exhibits weak correlations with the 2 migration indices (*r* = 0.127, and 0.132).
- On Axis 1, mean body mass (also relatively close to the origin) represents a contrast with all 4 prevalence indices (*r* = −0.2861, −0.2628, −0.2658 and −0.1866). The same is true for the mean abundance of *Trochiliphagus* (*r* = −0.2808). These results indicate relatively weak negative covariations.
- Axis 2 is dominated by a dichotomy between mean elevation and climatic indices, the mean, and the predictability of temperature and precipitation. The high negative correlation between elevation and temperature (*r* = −0.5451) is an expected observation.
- The 2 indices for migration behaviour are very close to each other (*r* = 0.962). Both are located close to the infestation indices, suggesting some positive interaction between them, the correlations ranging from 0.234 to 0.323.
- Considering the 2 different prevalence indices (without or with eggs also considered) for all lice (pooled), they are very close (*r* = 0.959). This means that the information carried by these 2 indices is roughly similar.
- The infestation indices (2 types of prevalence and abundance) referring to all lice (pooled) are positioned close to the prevalence and abundance of *Trochiloecetes* (correlations ranging from 0.615 to 0.958 for prevalence and 0.51 to 0.565 for abundance), but more distant from *Trochiliphagus* infestation indices. This simply means that the former genus was more commonly collected than the latter.
- Prevalence and abundance of *Trochiliphagus* are close to mean elevation, reflecting a positive correlation between them (*r* = 0.3294 and 0.4202).

The correlation matrix is provided in Additional file 1: Table S8.

## Discussion

There are 4 described species of *Leremenopon* from 7 species of hummingbirds (Dalgleish and Price, 2003*a*). Recently, Oniki-Willis *et al.* (2023) mentioned the occurrence of eggs of *Leremenopon* from 74 other species and subspecies of hummingbirds. The present record from *C. urochrysia* is a new host record for *Leremenopon* and also the first case of any identified louse from *C. urochrysia*.

There are 3 species of *Myrsidea* described from 3 species of hummingbirds (Dalgleish and Price, 2003*b*). In addition, unidentified specimens of *Myrsidea* from 6 other species of hummingbird have been mentioned (Dalgleish and Price, 2003*b*; Silva, 2013; Soto-Patiño *et al.*, 2018). The present observation from *P. longirostris* is a new host record for this genus.

There are 13 described species of *Trochiliphagus* from 14 species of hummingbirds (Price *et al.*, 2003). Oniki-Willis *et al.* (2023) mentioned the occurrence of *Trochiliphagus* eggs or nits from 174 other species or subspecies of hummingbirds, including all 10 species infested by *Trochiliphagus* in the present study (Table 2). These records represent the first cases of any identified louse from *Cynanthus canivetii, Eupherusa eximia, E. nigriventris, E. chionogaster, Heliodoxa jacula, Lampornis hemileucus* and *P. striigularis*. Generally, *Trochiliphagus* infestations are rarely found. Oniki-Willis *et al.* (2023) found no eggs on large samples of *Metallura tyrianthina*, *Lesbia victoriae*, *Aglaeactis cupripennis* and *Coeligena torquata* (*n* = 687, 408, 344, 316, respectively). Despite large sample size, *Trochiliphagus* was not found on *Campylopterus hemileucurus* (*n* = 43 in the present, and 276 in their study).

There are 30 described species of *Trochiloecetes* from 29 species of hummingbirds (Price *et al.*, 2003). Oniki-Willis *et al.* (2023) reported the occurrence of eggs from another 223 species of hummingbirds, including the 15 species infested with *Trochiloecetes* in the present study (Table 2). These are the first cases of any identified louse from *C. urochrysia*, *E. eximia*, *E. nigriventris*, *E. chionogaster*, *P. insignis*, *Lampornis calolaemus*, *L. cinereicauda*, *Microchera cupreiceps*, *Phaethornis atrimentalis* and *Threnetes ruckeri.*

There are no records of lice infesting some species of hummingbirds, even where large sample sizes have been examined. For example, Oniki-Willis *et al.* (2023) found no louse eggs on large samples of *Colibri coruscans*, *C. thalassinus*, and *C. delphinae* (*n* = 769, 370, 250, respectively). On the other hand, *Trochiloecetes latitemporalis* was described from *C. coruscans* (Carriker, 1960), although this was based on 1 individual. Similarly to Oniki-Willis *et al.* (2023), no louse or louse eggs were found on *C. cyanotus, Doryfera johannae* and *D. ludovicae* in the present study. Formerly, 1 specimen of *Trochiliphagus* was reported from *D. ludovicae* (Clayton *et al.*, 1992).

**Figure 3.**
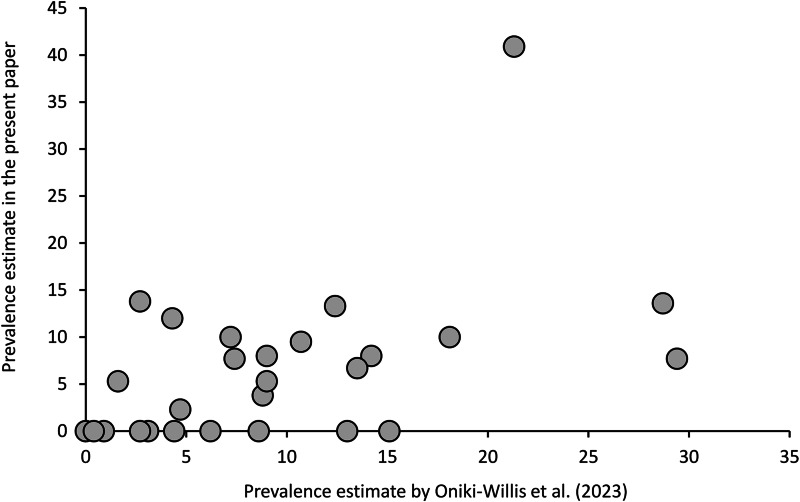
*Trochiloecetes* and *Trochiliphagus* prevalence estimates by Oniki-Willis *et al.* (2023) correlate strongly with the present estimates (samples with *n* < 10 were excluded) indicating a good repeatability of these estimates through space and time.

Since they applied rather different methods, data of Oniki-Willis *et al.* (2023) are not fully comparable to our results; however, the documented patterns are roughly similar. Overall, their estimates of prevalence are strongly correlated with the present estimates, after excluding samples with *n* < 10 (Fig. 3, Additional file 1: Table S7). Given that the former authors used a very different approach (much larger sample sizes, but unknown collection sites and dates, detecting eggs only, etc.), repeatability of estimates of prevalence is surprisingly good [Pearson's correlation: *r*(27) = 0.495, *P* = 0.0087].

There is only 1 published record of *Trochiliphagus* and *Trochiloecetes* co-occurring on the same host individual from *S. flammula* (Carriker, 1903). Coincidentally, 1 co-infested individual is reported here from the same host species. This does not contradict Oniki-Willis *et al.* (2023), who found that in very large samples of hummingbird species, the co-occurrence of eggs on the same birds was more frequent than expected by chance. First, the co-occurrence of eggs on the museum skins does not necessarily mean that the 2 infestations were simultaneous, as they might have accumulated in different periods. Second, given the rarity of these lice, the present sample sizes per host species are too small to test whether co-infestations are either more or less frequent than expected by chance.

Most infested hummingbirds were parasitized with only 1–10 lice. One notable exception was a female of *Campylopterus hemileucurus*, which hosted 33 *Trochiloecetes* (14 females, 7 males, 12 nymphs). They are relatively large lice (1.5–2.5 mm), about the size of the human louse *Pediculus humanus*. Based on the weight of human lice (0.0005 g, Speare *et al.*, 2006), the weight of the 33 *Trochiloecetes* specimens can be estimated at 0.2% of the host's body weight (9.5 g). Considering that these parasites are haematophagous and may also have a still unknown vector potential (Nelson, 1972), such high levels of infestation may negatively influence host condition.

We observed an unexpected positive covariation between mean elevation and the prevalence and abundance of *Trochiliphagus*. Since bird body mass was unrelated to elevation, this covariation is not rooted in differences in body mass at different elevations. Gustafsson *et al.* (2019) also reported an unusually high prevalence of lice on small passerines at high geographic elevations. They suggested that this may reflect differences in environmental factors such as ambient relative humidity, which is known to affect some louse assemblages (Bush *et al.*, 2009). On the other hand, relative humidity had little effect on avian louse communities in the Azores (Ošlejšková *et al.*, 2020). Since high-elevation habitats may provide more limited food resources to hummingbirds than low-elevation ones, aggressive interactions with body-to-body contacts (both within and between species) may be more frequent, providing more opportunities for horizontal transmission of lice (Johnson and Clayton, 2003) at high elevations. This may be analogous to the effect of forming mixed species feeding flocks in passerines (Gustafsson *et al.*, 2019). Alternatively, birds' energy constraints may be stricter at higher elevations so that birds may allocate less time and energy to antiparasitic behaviours like preening.

Both indices of migratory behaviour correlated with infestation indices, indicating that migrating birds had a higher prevalence and abundance of lice. This contrasts with former reports (Literák *et al.*, 2015; Sychra *et al.*, 2023) where there were higher prevalences of infestation in resident or short-distance migrants than in long-distance ones among European passerines. However, migration in hummingbirds examined in our study is of a different nature, as they most often exhibit elevational migration rather than latitudinal. Similarly, elevational migrant passerines have more prevalent infections by blood parasites than resident ones (Ishtiaq and Barve, 2018). The causes of the interaction between hummingbird migration and infestation indices are not yet understood.

Host body mass negatively correlated with infestation indices. This weak relationship was most clearly expressed in the prevalence of all lice (pooled as if they constituted 1 ecological guild), whether eggs were considered as a sign of infestation or not. This was surprising because the opposite tendency usually applies to avian lice (Rothschild and Clay, 1952; Rózsa, 1997). Note that former authors (Oniki-Willis *et al.*, 2023) found no effect of host body mass on infestations of hummingbird lice, indicating that there might be no relationship of the usual positive host mass/parasite indices in this particular host–parasite system. The reasons for this are unknown.

Former authors working with ricinid lice (Carriker, 1960; Rheinwald, 1968; Nelson, 1972) reported strongly female-biased sex ratios. This was verified in the case of *Trochiloecetes* in our sample. This was not the case for *Trochiliphagus*, in which sex ratio was less biased, probably because this genus was encountered so infrequently.

Overall, multivariate analysis using the d-correlation method, which allows evaluation of variables with different scale types, provides a useful approach to studying the ecology of louse communities. This method revealed several formerly unreported differences in the ecological characteristics and infestation measures of *Trochiliphagus* and *Trochiloecetes*. Former reports on this subject are limited to a very few papers. However, the general head and body structure of these lice indicate an analogy with the ‘ecotypes’ called ‘head lice’ and ‘body lice’ which emerged repeatedly during the evolution of ischnoceran lice (Johnson *et al.*, 2012; Bush *et al.*, 2016). *Trochiloecetes*, characterized by a large head and short oval body, occurs on the head and neck, while *Trochiliphagus*, characterized by a narrow, slender body, occurs on the host's body. The parallelism with ischnoceran ‘head lice’ and ‘body lice’ ecotypes indicates convergent evolution that can potentially help to explore their ecology. Factors affecting distribution and abundance of *Trochiloecetes* are almost unknown. However, *Trochiliphagus* is closely related to species of *Ricinus*, which infest mainly small passerines. Future studies will show whether the current knowledge of *Ricinus* ecology (Rheinwald, 1968; Nelson, 1972) may apply to them or not.

## Supporting information

Sychra et al. supplementary material Sychra et al. supplementary material

## Data Availability

Data generated or analysed during this study are included in this published article (and its supplementary information files). Complete datasets are available from the corresponding authors on reasonable request.

## Supplementary material

The supplementary material for this article can be found at https://doi.org/10.1017/S0031182023001294
