# Supplementary material for: Multivariate study of lice (Insecta: Psocodea: Phthiraptera) assemblages hosted by hummingbirds (Aves: Trochilidae)
Source: Parasitology. 2023 Dec 20;151(2):191–9. doi: 10.1017/S0031182023001294 (PMC10941040; doi:10.1017/S0031182023001294)
Supplement: Sychra et al. supplementary material [file S0031182023001294sup001.docx]

**Multivariate study of lice (Insecta: Psocodea: Phthiraptera) assemblages hosted by hummingbirds (Aves: Trochilidae)**

**Oldřich Sychra^1^*****, Lajos Rózsa^2,3,4^*****, János Podani^2,5^, Vojtěch Sychra^1^, Ivan Literák^1^ and Miroslav Capek^6^**

^1^ Department of Biology and Wildlife Diseases, Faculty of Veterinary Hygiene and Ecology, University of Veterinary Sciences, Brno, Czechia

^2^ Institute of Evolution, ELKH Centre for Ecological Research, Budapest, Hungary

^3^ Centre for Eco-Epidemiology, National Laboratory for Health Security, Budapest, Hungary

^4^ Hungarian Department of Biology and Ecology, Babeș-Bolyai University, Cluj-Napoca, Romania

^5^ Department of Plant Systematics, Ecology and Theoretical Biology, Institute of Biology, Eötvös University, Budapest, Hungary

^6^ Institute of Vertebrate Biology, Czech Academy of Sciences, Brno, Czechia

*Correspondence: sychrao@vfu.cz, lajos.rozsa@gmail.com; Oldřich Sychra and Lajos Rózsa contributed equally to this work.

**ELECTRONIC SUPPLEMENTARY MATERIAL**

**Methods**

**Additional file 1: Text S1.** We evaluated, modified and updated data about the body mass of males and females, the sexual dichromatism and migration behavior in Diamant *et al.* (2021) by studying the following publications:

**Angehr GR, and Dean R** (2010) *The Birds of Panama: A Field Guide.* Cornell University Press, Ithaca & London.

**Arlott N, van Perlo B, Carrizo G, Chiappe AA, Huber L, Mata JRR, Erize F, de la Peña MR, Sharp C and Straneck R** (2021) *Collins Birds of the World.* HarperCollins, London.

**Ascanio D, Rodriguez G and Restall R** (2017) *Birds of Venezuela.* Christopher Helm, London.

**del Hoyo J** **(ed.)** (2020) *All the Birds of the World.* Lynx Edicions, Barcelona.

**del Hoyo J and Collar NJ** (2014) *HBW and Birdlife International Illustrated Checklist of the Birds of the World. Vol. 1: Non-Passerines*. Lynx Edicions, Barcelona.

**del Hoyo J, Elliott A and Sargatal J (eds.)** (1999) *Handbook of the Birds of the World. Vol. 5: Barn-owls to Hummingbirds.* Lynx Edicions, Barcelona.

**Fagan J and Komar O** (2016) *Peterson Field Guide to Birds of Northern Central America.* Houghton Mifflin Harcourt, Boston & New York.

**Fogden M, Taylor M and Williamson SL** (2004) *Hummingbirds. A Life-size Guide to Every Species.* Ivy Press, Lewes.

**Gallardo RJ** (2014) *Guide to the Birds of Honduras.* Mountain-Gem Bird Tours, Tegucigalpa.

**Garrigues R and Dean R** (2014) *The Birds of Costa Rica: A Field Guide.* Cornell University Press, Ithaca & London.

**Gill F, Donsker D and Rasmussen P (eds)** (2023) *IOC World Bird List (v 13.2).* Doi 10.14344/IOC.ML.13.2. (accessed 30 Aug 2023)

**Hilty SL and Brown WL** (1986) *A Guide to the Birds of Colombia*. Princeton University Press, Princeton & Guildford.

**Hilty SL** (2021) *Birds of Colombia.* Lynx and BirdLife International Field Guides. Lynx Edicions, Barcelona.

**Jones HL** (2003) *Birds of Belize.* University of Texas Press, Austin.

**McMullan M** (2018) *Field Guide to the Birds of Colombia*. Rey Narajo Editores, Bogota.

**Quiñones FA** (2018) *Guia ilustrada de la Avifauna colombiana* [*A Field Guide to the Birds of Colombia*]. Wildlife Conservation Society, Cali.

**Restall R, Rodner C and Lentino M** (2006) *Birds of Northern South America. An Identification Guide. Vol. 1. Species Accounts.* Christopher Helm, London.

**Restall R, Rodner C and Lentino M** (2006) *Birds of Northern South America. An Identification Guide. Vol. 2. Plates and Maps.* Christopher Helm, London.

**Ridgely RS and Greenfield PJ** (2001) *The Birds of Ecuador. Vol. 2.* Christopher Helm, London.

**Ridgely RS, Gwynne JA, Tudor G and Argel M** (2016) *Wildlife Conservation Society Birds of Brazil: The Atlantic Forest of Southeast Brazil, including São Paulo and Rio de Janeiro.* Cornell University Press, Ithaca.

**Schulenberg TS, Stotz DE, Lane DE, O’Neill JP and Parker TA, III.** (2010) *Birds of Peru.* Rev. ed. Princeton University Press. Princeton & Oxford.

**Stiles FG and Skutch AF** (1989) *A Guide to the Birds of Costa Rica.* Cornell University Press, Ithaca & New York.

**Vallely AC and Dyer D** (2018) *Birds of Central America: Belize, Guatemala, Honduras, El Salvador, Nicaragua, Costa Rica, and Panama.* Princeton University Press, Princeton & Oxford.

**Additional file 1: Table S1.** List of locations with coordinates, elevation and date of collection of lice from hummingbirds in Central and South America.

| **Country** | **Abbreviation** | **Location** | **Coordinates** | **Elevation**  **(m a.s.l.)** | **Date** |
| --- | --- | --- | --- | --- | --- |
| Honduras | Ho_LA | Atlántida, Tela, Botanical Garden Lancetilla | 15°44' N, 87°27' W | 256 | 10.–18.8.2014 |
| Honduras | Ho_UT | Islas de la Bahía, Utila | 16°06' N, 86°54' W | 4 | 22.–30.8.2014 |
| Costa Rica | CR_HC | Hitoy Cerere Biological Reserve, Cordillera de Talamanca mountain range, Provincia Limón | 9°40' N, 83°01' W | 141 | 17.–31.8.2004 |
| Costa Rica | CR_B | Barbilla NP, Cordillera de Talamanca mountain range, Provincia Limón | 9°59' N, 83°25' W | 520 | 2.–11.9.2004 |
| Costa Rica | CR_RV | Rincón de la Vieja NP (sector Santa Maria), Cordillera de Guanacaste | 10°46' N, 85°18' W | 934 | 15.–24.8.2009 |
| Costa Rica | CR_T | Tapantí NP (sector Tapantí), Cordillera de Talamanca mountain range | 9°46' N, 83°47' W | 1333 | 31.7.–11.8.2009 |
| Costa Rica | CR_LT | Zona Protectora Las Tablas | 8°54' N, 82°47' W | 1413 | 18.–22.8.2010 |
| Costa Rica | CR_BV | Braulio Carrillo NP (sector Barva) | 10°07' N, 84°07' W | 2229 | 30.7.–8.8.2010 |
| Costa Rica | CR_CM | Tapantí NP (sector Cerro de la Muerte) | 9°33' N, 83°43' W | 2987 | 11.–15.8.2010 |
| Peru | Pe_HU | Cascay, Provincia de Huánuco | 22°31' S, 53°30' W | 2365 | 20.–23.8.2011 |
| Peru | Pe_TR | Centro Urku, Tarapoto | 6°27' S, 76°21' W | 779 | 8.–10.8.2011 |
| Peru | Pe_IQ | Reserva Nacional Allpahuayo Mishana, Iquitos | 3°58' S, 73°25' W | 128 | 13.–16.8.2011 |
| Peru | Pe_PV | Refugio de Vida Silvestre Los Pantanos de Villa, Lima | 12°13' S, 76°59' W | 3 | 19.–22.7.2011 |
| Brazil | B_FBD&MI | Fazenda Belcanto and farma Mauro near Ivinhema River, Mato Grosso do Sul state | 22°31' S, 53°30' W | 248 | 12.8.+15.–16.8.2006 |
| Brazil | B_NA | Nova Andranina, Mato Grosso do Sul state | 22°15' S, 53°21' W | 370 | 16.–17.7.+29.7.–12.8.2006 |
| Brazil | B_P | Margarida at the foothills of the Cerra de Bodoquena, Mato Grosso do Sul state in Brazilian Pantanal | 21°30' S, 56°40' W | 428 | 20.–21.7.2006 |
| Paraguay | PG_AE | Teniente Agripino Enciso NP | 21°12' S, 61°39' W | 256 | 31.8.–3.9.2012 |
| Paraguay | PG_SR | San Rafael NP | 26°30' S, 55°47' W | 168 | 18.–24.8.2012 |
| Paraguay | PG_TG | Los Tres Gigantes Biological Station in the Paraguayan Pantanal | 20°04' S, 58°09' W | 82 | 6.–9.9.2012 |

**Additional file 1: Table S2.** List of hummingbirds examined at locations in Brazil, Costa Rica, Honduras, Paraguay and Peru.

Numbers of birds parasitized, including the presence of louse eggs / examined birds are included.

For abbreviations of locations, see Additional file 1: Table S1.

|  | **Honduras** | | **Costa Rica** | | | | | | | **Peru** | | | | **Brazil** | | **Paraguay** | | |  |
| --- | --- | --- | --- | --- | --- | --- | --- | --- | --- | --- | --- | --- | --- | --- | --- | --- | --- | --- | --- |
| **Location** | **LA** | **UT** | **HC** | **B** | **RV** | **T** | **LT** | **BV** | **CM** | **HU** | **TR** | **IQ** | **PV** | **NA** | **P** | **AE** | **SR** | **TG** | **Total** |
| **Elevation (m a.s.l.)** | **256** | **4** | **141** | **520** | **934** | **1333** | **1413** | **2229** | **2987** | **2365** | **779** | **128** | **3** | **248** | **370** | **428** | **256** | **168** |  |
| tribes/spp. hummingbirds |  |  |  |  |  |  |  |  |  |  |  |  |  |  |  |  |  |  |  |
| **Hermits (Phaethornithinae)** |  |  |  |  |  |  |  |  |  |  |  |  |  |  |  |  |  |  |  |
| *Eutoxeres aquila salvini* Gould, 1868 |  |  | 0/1 | 0/11 |  | 0/1 |  |  |  |  |  |  |  |  |  |  |  |  | 0/13 |
| *Glaucis aeneus* Lawrence, 1868 |  |  | 0/9 | 0/1 |  |  |  |  |  |  |  |  |  |  |  |  |  |  | 0/10 |
| *Glaucis hirsutus hirsutus* (Gmelin, JF, 1788) |  |  |  |  |  |  |  |  |  |  | 0/2 | 0/3 |  |  |  |  |  |  | 0/5 |
| *Phaethornis atrimentalis atrimentalis* Lawrence, 1858 |  |  |  |  |  |  |  |  |  |  | 2/4 |  |  |  |  |  |  |  | 2/4 |
| *Phaethornis eurynome paraguayensis* Bertoni, MS & Bertoni, AW, 1901 |  |  |  |  |  |  |  |  |  |  |  |  |  |  |  |  | 0/3 |  | 0/3 |
| *Phaethornis guy coruscus* Bangs, 1902 |  |  |  |  |  | 0/35 | 0/8 |  |  |  |  |  |  |  |  |  |  |  | 0/43 |
| *Phaethornis longirostris baroni* Hartert, EJO, 1897 |  |  |  |  |  |  |  |  |  |  | 1/5 | 1/5 |  |  |  |  |  |  | 2/10 |
| *Phaethornis longirostris cephalus* (Bourcier & Mulsant, 1848) |  |  | 0/27 | 0/11 |  |  |  |  |  |  |  |  |  |  |  |  |  |  | 0/38 |
| *Phaethornis longirostris longirostris* (Delattre, 1843) | 9/27 |  |  |  |  |  |  |  |  |  |  |  |  |  |  |  |  |  | 9/27 |
| *Phaethornis pretrei* (Lesson, RP & Delattre, 1839) |  |  |  |  |  |  |  |  |  |  |  |  |  | 0/2 |  |  |  |  | 0/2 |
| *Phaethornis striigularis saturatus* Ridgway, 1910 | 0/2 |  | 0/9 |  | 2/2 | 0/2 |  |  |  |  |  |  |  |  |  |  |  |  | 2/15 |
| *Threnetes leucurus cervinicauda* Gould, 1855 |  |  |  |  |  |  |  |  |  |  | 1/3 |  |  |  |  |  |  |  | 1/3 |
| *Threnetes ruckeri ventosus* Bangs & Penard, TE, 1924 | 1/1 |  | 1/12 | 3/17 |  |  |  |  |  |  |  |  |  |  |  |  |  |  | 5/30 |
| **Mangoes (Polythminae)** |  |  |  |  |  |  |  |  |  |  |  |  |  |  |  |  |  |  |  |
| *Anthracothorax prevostii gracilirostris* Ridgway, 1910 | 1/1 | 0/2 |  |  |  |  |  |  |  |  |  |  |  |  |  |  |  |  | 1/3 |
| *Colibri cyanotus cabanidis* (Heine, 1863) |  |  |  |  |  | 0/1 |  | 0/3 | 0/2 |  |  |  |  |  |  |  |  |  | 0/6 |
| *Doryfera johannae johannae* (Bourcier, 1847) |  |  |  |  |  |  |  |  |  |  | 0/1 |  |  |  |  |  |  |  | 0/1 |
| *Doryfera ludovicae veraguensis* Salvin, 1867 |  |  |  |  |  | 0/1 |  |  |  |  |  |  |  |  |  |  |  |  | 0/1 |
| *Heliothryx barroti* (Bourcier, 1843) |  |  |  |  |  |  | 0/1 |  |  |  |  |  |  |  |  |  |  |  | 0/1 |
| **Brilliants (Lesbiinae – Coeligenini)** |  |  |  |  |  |  |  |  |  |  |  |  |  |  |  |  |  |  |  |
| *Heliodoxa jacula henryi* Lawrence, 1867 |  |  |  |  |  | 1/10 |  |  |  |  |  |  |  |  |  |  |  |  | 1/10 |
| **Mtn. Gems (Trochilinae – Lampornini)** |  |  |  |  |  |  |  |  |  |  |  |  |  |  |  |  |  |  |  |
| *Eugenes spectabilis* (Lawrence, 1867) |  |  |  |  |  |  |  | 0/1 | 5/25 |  |  |  |  |  |  |  |  |  | 5/26 |
| *Heliomaster constantii constantii* (Delattre, 1843) |  |  |  |  | 0/7 |  |  |  |  |  |  |  |  |  |  |  |  |  | 0/7 |
| *Lampornis calolaemus calolaemus* (Salvin, 1865) |  |  |  |  |  | 0/1 |  | 7/28 |  |  |  |  |  |  |  |  |  |  | 7/29 |
| *Lampornis cinereicauda* (Lawrence, 1867) |  |  |  |  |  |  |  |  | 2/5 |  |  |  |  |  |  |  |  |  | 2/5 |
| *Lampornis hemileucus* (Salvin, 1865) |  |  |  |  |  | 1/8 |  |  |  |  |  |  |  |  |  |  |  |  | 1/8 |
| *Panterpe insignis insignis* Cabanis & Heine, 1860 |  |  |  |  |  |  |  | 0/1 | 5/41 |  |  |  |  |  |  |  |  |  | 5/42 |
| **Bees (Trochilinae – Mellisugini)** |  |  |  |  |  |  |  |  |  |  |  |  |  |  |  |  |  |  |  |
| *Selasphorus flammula simoni* Carriker, 1910 |  |  |  |  |  |  |  | 8/12 |  |  |  |  |  |  |  |  |  |  | 8/12 |
| *Selasphorus flammula torridus* Salvin, 1870 |  |  |  |  |  |  |  |  | 5/10 |  |  |  |  |  |  |  |  |  | 5/10 |
| **Emeralds (Trochilinae – Cynanthini)** |  |  |  |  |  |  |  |  |  |  |  |  |  |  |  |  |  |  |  |
| *Campylopterus hemileucurus mellitus* Bangs, 1902 |  |  |  |  |  | 1/35 | 0/5 | 0/3 |  |  |  |  |  |  |  |  |  |  | 1/43 |
| *Cynanthus canivetii salvini* (Cabanis & Heine, 1860) |  | 0/4 |  |  | 1/3 |  |  |  |  |  |  |  |  |  |  |  |  |  | 1/7 |
| *Chlorostilbon lucidus lucidus* (Shaw, 1812) |  |  |  |  |  |  |  |  |  |  |  |  |  | 0/1 | 0/1 | 0/2 |  |  | 0/4 |
| *Klais guimeti merrittii* (Lawrence, 1860) |  |  |  |  | 0/2 | 0/1 |  |  |  |  |  |  |  |  |  |  |  |  | 0/3 |
| *Stephanoxis loddigesii* (Vigors, 1831) |  |  |  |  |  |  |  |  |  |  |  |  |  |  |  |  | 0/1 |  | 0/1 |
| **Emeralds/Amazilias (Trochilinae – Trochilini)** |  |  |  |  |  |  |  |  |  |  |  |  |  |  |  |  |  |  |  |
| *Amazilia rutila corallirostris* (Bourcier & Mulsant, 1846) |  |  |  |  | 0/9 |  |  |  |  |  |  |  |  |  |  |  |  |  | 0/9 |
| *Amazilia tzacatl tzacatl* (de la Llave, 1833) | 2/33 |  | 1/9 | 0/4 |  |  | 1/4 |  |  |  |  |  |  |  |  |  |  |  | 4/50 |
| *Amazilis amazilia amazilia* (Lesson, RP & Garnot, 1827) |  |  |  |  |  |  |  |  |  |  |  |  | 0/1 |  |  |  |  |  | 0/1 |
| *Elliotomyia chionogaster chionogaster* (Tschudi, 1846) |  |  |  |  |  |  |  |  |  | 4/19 |  |  |  |  |  |  |  |  | 4/19 |
| *Eupherusa eximia egregia* Sclater, PL & Salvin, 1868 |  |  |  |  | 3/6 | 1/1 |  | 1/1 |  |  |  |  |  |  |  |  |  |  | 5/8 |
| *Eupherusa nigriventris* Lawrence, 1868 |  |  |  |  |  | 3/5 |  |  |  |  |  |  |  |  |  |  |  |  | 3/5 |
| *Hylocharis chrysura* (Shaw, 1812) |  |  |  |  |  |  |  |  |  |  |  |  |  |  |  | 0/2 | 0/4 | 0/1 | 0/7 |
| *Chalybura urochrysia melanorrhoa* Salvin, 1865 |  |  | 2/13 | 1/7 |  |  |  |  |  |  |  |  |  |  |  |  |  |  | 3/20 |
| *Chionomesa fimbriata laeta* (Hartert, EJO, 1900) |  |  |  |  |  |  |  |  |  |  |  | 0/1 |  |  |  |  |  |  | 0/1 |
| *Chionomesa lactea bartletti* (Gould, 1866) |  |  |  |  |  |  |  |  |  |  | 0/2 |  |  |  |  |  |  |  | 0/2 |
| *Chlorestes candida candida* (Bourcier & Mulsant, 1846) | 2/13 |  |  |  |  |  |  |  |  |  |  |  |  |  |  |  |  |  | 2/13 |
| *Chlorestes eliciae eliciae* (Bourcier & Mulsant, 1846) | 0/2 |  |  |  |  |  |  |  |  |  |  |  |  |  |  |  |  |  | 0/2 |
| *Microchera cupreiceps* (Lawrence, 1866) |  |  |  |  | 1/1 |  |  |  |  |  |  |  |  |  |  |  |  |  | 1/1 |
| *Microchera chionura* (Gould, 1851) |  |  |  |  |  |  | 0/1 |  |  |  |  |  |  |  |  |  |  |  | 0/1 |
| *Phaeochroa cuvierii maculicauda* Griscom, 1932 |  |  |  |  |  |  | 0/3 |  |  |  |  |  |  |  |  |  |  |  | 0/3 |
| *Polyerata amabilis* (Gould, 1853) |  |  | 0/4 |  |  |  |  |  |  |  |  |  |  |  |  |  |  |  | 0/4 |
| *Saucerottia edward niveoventer* (Gould, 1851) |  |  |  |  |  |  | 0/1 |  |  |  |  |  |  |  |  |  |  |  | 0/1 |
| *Saucerottia hoffmanni* (Cabanis & Heine, 1860) |  |  |  |  | 0/4 |  |  |  |  |  |  |  |  |  |  |  |  |  | 0/4 |
| *Thalurania colombica townsendi* Ridgway, 1888 | 0/2 |  |  |  |  |  |  |  |  |  |  |  |  |  |  |  |  |  | 0/2 |
| *Thalurania colombica venusta* (Gould, 1851) |  |  | 0/2 |  | 0/1 |  |  |  |  |  |  |  |  |  |  |  |  |  | 0/3 |
| *Thalurania furcata viridipectus* Gould, 1848 |  |  |  |  |  |  |  |  |  |  |  | 0/1 |  |  |  |  |  |  | 0/1 |
| **Total** | **15/81** | **0/6** | **4/86** | **4/51** | **7/35** | **7/101** | **1/23** | **16/49** | **17/83** | **4/19** | **4/17** | **1/10** | **0/1** | **0/3** | **0/1** | **0/4** | **0/8** | **0/1** | **80/579** |

**Additional file 1: Table S3.** Prevalence of chewing lice on hummingbirds according to the sex of hosts and their colour dimorphism, i.e., 1) dimorphic in colour – species with conspicuous differences in colour between males and females (33 species); 2) monomorphic in colour – species without or only with slight differences in colour between males and females (16 species). Undetermined sex = impossible to distinguish the sex, i.e., young male/female/moulting bird or monomorphic species.

E=number of examined birds, P=number of birds parasitized with lice, Eg=number of birds in which louse eggs were found, PEg=number of birds in which lice and/or louse eggs were found, Ego=number of birds in which only louse eggs and no lice were found.

|  |  | **Female** | | | | **Male** | | | | **Undetermined** | | | | **TOTAL** | | | | |
| --- | --- | --- | --- | --- | --- | --- | --- | --- | --- | --- | --- | --- | --- | --- | --- | --- | --- | --- |
|  | **Colour dimorphism** | **E** | **P** | **Eg** | **PEg** | **E** | **P** | **Eg** | **PEg** | **E** | **P** | **Eg** | **PEg** | **E** | **P** | **Eg** | **PEg** | **Ego** |
| **Hermits (Phaethornithinae)** |  |  |  |  |  |  |  |  |  |  |  |  |  |  |  |  |  |  |
| *Eutoxeres aquila* | monomorphic |  |  |  |  |  |  |  |  | 13 |  |  |  | 13 |  |  |  |  |
| *Glaucis aeneus* | dimorphic |  |  |  |  |  |  |  |  | 10 |  |  |  | 10 |  |  |  |  |
| *Glaucis hirsutus* | dimorphic |  |  |  |  |  |  |  |  | 5 |  |  |  | 5 |  |  |  |  |
| *Phaethornis atrimentalis* | dimorphic |  |  |  |  |  |  |  |  | 4 | 2 | 2 | 2 | 4 | 2 | 2 | 2 |  |
| *Phaethornis eurynome* | monomorphic |  |  |  |  |  |  |  |  | 3 |  |  |  | 3 |  |  |  |  |
| *Phaethornis guy* | dimorphic | 33 |  |  |  | 8 |  |  |  | 2 |  |  |  | 43 |  |  |  |  |
| *Phaethornis longirostris* | monomorphic |  |  |  |  |  |  |  |  | 75 | 10 | 2 | 11 | 75 | 10 | 2 | 11 | 1 |
| *Phaethornis pretrei* | monomorphic |  |  |  |  |  |  |  |  | 2 |  |  |  | 2 |  |  |  |  |
| *Phaethornis striigularis* | monomorphic |  |  |  |  |  |  |  |  | 15 | 1 | 1 | 2 | 15 | 1 | 1 | 2 | 1 |
| *Threnetes leucurus* | dimorphic |  |  |  |  |  |  |  |  | 3 | 1 |  | 1 | 3 | 1 |  | 1 |  |
| *Threnetes ruckeri* | monomorphic |  |  |  |  |  |  |  |  | 30 | 4 | 3 | 5 | 30 | 4 | 3 | 5 | 1 |
| **Mangoes (Polythminae)** |  |  |  |  |  |  |  |  |  |  |  |  |  |  |  |  |  |  |
| *Anthracothorax prevostii* | dimorphic |  |  |  |  |  |  |  |  | 3 |  | 1 | 1 | 3 |  | 1 | 1 | 1 |
| *Colibri cyanotus* | monomorphic |  |  |  |  |  |  |  |  | 6 |  |  |  | 6 |  |  |  |  |
| *Doryfera johannae* | dimorphic |  |  |  |  |  |  |  |  | 1 |  |  |  | 1 |  |  |  |  |
| *Doryfera ludovicae* | dimorphic |  |  |  |  | 1 |  |  |  |  |  |  |  | 1 |  |  |  |  |
| *Heliothryx barroti* | dimorphic | 1 |  |  |  |  |  |  |  |  |  |  |  | 1 |  |  |  |  |
| **Brilliants (Lesbiinae – Coeligenini)** |  |  |  |  |  |  |  |  |  |  |  |  |  |  |  |  |  |  |
| *Heliodoxa jacula* | dimorphic | 9 | 1 | 1 | 1 | 1 |  |  |  |  |  |  |  | 10 | 1 | 1 | 1 |  |
| **Mtn. Gems (Trochilinae – Lampornini)** |  |  |  |  |  |  |  |  |  |  |  |  |  |  |  |  |  |  |
| *Eugenes spectabilis* | dimorphic | 6 |  |  |  | 11 | 3 | 3 | 4 | 9 |  | 1 | 1 | 26 | 3 | 4 | 5 | 2 |
| *Heliomaster constantii* | dimorphic |  |  |  |  |  |  |  |  | 7 |  |  |  | 7 |  |  |  |  |
| *Lampornis calolaemus* | dimorphic | 11 | 2 | 2 | 3 | 9 |  |  |  | 9 | 2 | 4 | 4 | 29 | 4 | 6 | 7 | 3 |
| *Lampornis cinereicauda* | dimorphic | 3 | 2 | 1 | 2 | 2 |  |  |  |  |  |  |  | 5 | 2 | 1 | 2 |  |
| *Lampornis hemileucus* | dimorphic | 7 | 1 |  | 1 |  |  |  |  | 1 |  |  |  | 8 | 1 |  | 1 |  |
| *Panterpe insignis* | monomorphic |  |  |  |  |  |  |  |  | 42 | 4 | 3 | 5 | 42 | 4 | 3 | 5 | 1 |
| **Bees (Trochilinae – Mellisugini)** |  |  |  |  |  |  |  |  |  |  |  |  |  |  |  |  |  |  |
| *Selasphorus flammula* | dimorphic |  |  |  |  | 4 | 3 | 3 | 3 | 18 | 8 | 8 | 10 | 22 | 11 | 11 | 13 | 2 |
| **Emeralds (Trochilinae – Cynanthini)** |  |  |  |  |  |  |  |  |  |  |  |  |  |  |  |  |  |  |
| *Campylopterus hemileucurus* | dimorphic | 16 | 1 |  | 1 | 19 |  |  |  | 8 |  |  |  | 43 | 1 |  | 1 |  |
| *Cynanthus canivetii* | dimorphic |  |  |  |  | 2 | 1 |  | 1 | 5 |  |  |  | 7 | 1 |  | 1 |  |
| *Chlorostilbon lucidus* | dimorphic |  |  |  |  |  |  |  |  | 4 |  |  |  | 4 |  |  |  |  |
| *Klais guimeti* | dimorphic | 1 |  |  |  |  |  |  |  | 2 |  |  |  | 3 |  |  |  |  |
| *Stephanoxis loddigesii* | dimorphic |  |  |  |  |  |  |  |  | 1 |  |  |  | 1 |  |  |  |  |
| **Emeralds/Amazilias (Trochilinae – Trochilini)** |  |  |  |  |  |  |  |  |  |  |  |  |  |  |  |  |  |  |
| *Amazilia rutile* | dimorphic |  |  |  |  |  |  |  |  | 9 |  |  |  | 9 |  |  |  |  |
| *Amazilia tzacatl* | dimorphic |  |  |  |  |  |  |  |  | 50 |  | 4 | 4 | 50 |  | 4 | 4 | 4 |
| *Amazilis amazilia* | monomorphic |  |  |  |  |  |  |  |  | 1 |  |  |  | 1 |  |  |  |  |
| *Elliotomyia chionogaster* | monomorphic |  |  |  |  |  |  |  |  | 19 | 2 | 2 | 4 | 19 | 2 | 2 | 4 | 2 |
| *Eupherusa eximia* | dimorphic | 1 |  |  |  | 6 | 2 | 3 | 4 | 1 | 1 |  | 1 | 8 | 3 | 3 | 5 | 2 |
| *Eupherusa nigriventris* | dimorphic | 1 |  |  |  | 1 | 1 | 1 | 1 | 3 | 2 | 2 | 2 | 5 | 3 | 3 | 3 |  |
| *Hylocharis chrysura* | monomorphic |  |  |  |  |  |  |  |  | 7 |  |  |  | 7 |  |  |  |  |
| *Chalybura urochrysia* | dimorphic | 2 |  |  |  | 2 |  |  |  | 16 | 3 | 1 | 3 | 20 | 3 | 1 | 3 |  |
| *Chionomesa fimbriata* | monomorphic |  |  |  |  |  |  |  |  | 1 |  |  |  | 1 |  |  |  |  |
| *Chionomesa lactea* | monomorphic |  |  |  |  |  |  |  |  | 2 |  |  |  | 2 |  |  |  |  |
| *Chlorestes candida* | monomorphic |  |  |  |  |  |  |  |  | 13 | 1 | 2 | 2 | 13 | 1 | 2 | 2 | 1 |
| *Chlorestes eliciae* | dimorphic |  |  |  |  |  |  |  |  | 2 |  |  |  | 2 |  |  |  |  |
| *Microchera cupreiceps* | dimorphic |  |  |  |  | 1 | 1 | 1 | 1 |  |  |  |  | 1 | 1 | 1 | 1 |  |
| *Microchera chionura* | dimorphic | 1 |  |  |  |  |  |  |  |  |  |  |  | 1 |  |  |  |  |
| *Phaeochroa cuvierii* | monomorphic |  |  |  |  |  |  |  |  | 3 |  |  |  | 3 |  |  |  |  |
| *Polyerata amabilis* | dimorphic | 2 |  |  |  | 2 |  |  |  |  |  |  |  | 4 |  |  |  |  |
| *Saucerottia edward* | monomorphic |  |  |  |  |  |  |  |  | 1 |  |  |  | 1 |  |  |  |  |
| *Saucerottia hoffmanni* | dimorphic |  |  |  |  |  |  |  |  | 4 |  |  |  | 4 |  |  |  |  |
| *Thalurania colombica* | dimorphic | 1 |  |  |  | 2 |  |  |  | 2 |  |  |  | 5 |  |  |  |  |
| *Thalurania furcata* | dimorphic |  |  |  |  |  |  |  |  | 1 |  |  |  | 1 |  |  |  |  |
| **Total number of birds** |  | 95 | 7 | 4 | 8 | 73 | 11 | 11 | 14 | 411 | 41 | 36 | 58 | 579 | 59 | 51 | 80 | 21 |
| **Number of species** |  | 15 | 4 | 2 | 4 | 16 | 6 | 5 | 6 | 42 | 13 | 14 | 16 | 49 | 19 | 18 | 22 | 12 |
| **Prevalence (%)** |  |  | 7.4 | 4.2 | **8.4** |  | 15.1 | 15.1 | **19.2** |  | 10.0 | 8.8 | **14.1** |  | 10.2 | 8.8 | **13.8** |  |
|  |  |  |  |  |  |  |  |  |  |  |  |  |  |  |  |  |  |  |
| Data for species with ≥ 10 individuals examined |  |  |  |  |  |  |  |  |  |  |  |  |  |  |  |  |  |  |
| **Total number of birds** |  | 77 | 4 | 3 | 5 | 54 | 6 | 6 | 7 | 329 | 35 | 31 | 51 | 460 | 45 | 40 | 63 | 18 |
| **Number of species** |  | 6 | 3 | 2 | 3 | 7 | 2 | 2 | 2 | 15 | 9 | 11 | 11 | 16 | 12 | 12 | 13 | 10 |
| **Prevalence (%)** |  |  | 5.2 | 3.9 | **6.5** |  | 11.1 | 11.1 | **13.0** |  | 10.6 | 9.4 | **15.5** |  | 9.8 | 8.7 | **13.7** |  |
|  |  |  |  |  |  |  |  |  |  |  |  |  |  |  |  |  |  |  |
| **Total number of birds** | dimorphic | 77 | 4 | 3 | 5 | 54 | 6 | 6 | 7 | 122 | 13 | 18 | 22 | 253 | 23 | 27 | 34 | 11 |
| **Number of species** | dimorphic | 6 | 3 | 2 | 3 | 7 | 2 | 2 | 2 | 8 | 3 | 5 | 5 | 9 | 6 | 6 | 7 | 4 |
| **Prevalence (%)** | dimorphic |  | 5.2 | 3.9 | **6.5** |  | 11.1 | 11.1 | **13.0** |  | 10.7 | 14.8 | **18.0** |  | 9.1 | 10.7 | **13.4** |  |
|  |  |  |  |  |  |  |  |  |  |  |  |  |  |  |  |  |  |  |
| **Total number of birds** | monomorphic |  |  |  |  |  |  |  |  |  |  |  |  | 207 | 22 | 13 | 29 | 7 |
| **Number of species** | monomorphic |  |  |  |  |  |  |  |  |  |  |  |  | 7 | 6 | 6 | 6 | 6 |
| **Prevalence (%)** | monomorphic |  |  |  |  |  |  |  |  |  |  |  |  |  | 10.6 | 6.3 | **14.0** |  |

**Additional file 1: Table S4.** Prevalence of chewing lice on hummingbirds from different elevations, i.e. 1) lowlands (0–550 m a.s.l.), 2) highlands (700–1500 m a.s.l.), 3) mountains (2000–3000 m a.s.l.)

E=number of examined birds, P=number of birds parasitized with lice, Eg=number of birds in which louse eggs were found, PEg=number of birds in which lice and/or louse eggs were found, Ego=number of birds in which only louse eggs and no lice were found.

|  |  | **Lowlands** | | | | **Highlands** | | | | **Mountains** | | | | **TOTAL** | | | |
| --- | --- | --- | --- | --- | --- | --- | --- | --- | --- | --- | --- | --- | --- | --- | --- | --- | --- |
|  | **Colour dimorphism** | **E** | **P** | **Eg** | **PEg** | **E** | **P** | **Eg** | **PEg** | **E** | **P** | **Eg** | **PEg** | **E** | **P** | **Eg** | **PEg** |
| **Hermits (Phaethornithinae)** |  |  |  |  |  |  |  |  |  |  |  |  |  |  |  |  |  |
| *Eutoxeres aquila* | monomorphic | 12 |  |  |  | 1 |  |  |  |  |  |  |  | 13 |  |  |  |
| *Glaucis aeneus* | dimorphic | 10 |  |  |  |  |  |  |  |  |  |  |  | 10 |  |  |  |
| *Glaucis hirsutus* | dimorphic | 3 |  |  |  | 2 |  |  |  |  |  |  |  | 5 |  |  |  |
| *Phaethornis atrimentalis* | dimorphic |  |  |  |  | 4 | 2 | 2 | 2 |  |  |  |  | 4 | 2 | 2 | 2 |
| *Phaethornis eurynome* | monomorphic | 3 |  |  |  |  |  |  |  |  |  |  |  | 3 |  |  |  |
| *Phaethornis guy* | dimorphic |  |  |  |  | 43 |  |  |  |  |  |  |  | 43 |  |  |  |
| *Phaethornis longirostris* | monomorphic | 70 | 9 | 2 | 10 | 5 | 1 |  | 1 |  |  |  |  | 75 | 10 | 2 | 11 |
| *Phaethornis pretrei* | monomorphic | 2 |  |  |  |  |  |  |  |  |  |  |  | 2 |  |  |  |
| *Phaethornis striigularis* | monomorphic | 11 |  |  |  | 4 | 1 | 1 | 2 |  |  |  |  | 15 | 1 | 1 | 2 |
| *Threnetes leucurus* | dimorphic |  |  |  |  | 3 | 1 |  | 1 |  |  |  |  | 3 | 1 |  | 1 |
| *Threnetes ruckeri* | monomorphic | 30 | 4 | 3 | 5 |  |  |  |  |  |  |  |  | 30 | 4 | 3 | 5 |
| **Mangoes (Polythminae)** |  |  |  |  |  |  |  |  |  |  |  |  |  |  |  |  |  |
| *Anthracothorax prevostii* | dimorphic | 3 |  | 1 | 1 |  |  |  |  |  |  |  |  | 3 |  | 1 | 1 |
| *Colibri cyanotus* | monomorphic |  |  |  |  | 1 |  |  |  | 5 |  |  |  | 6 |  |  |  |
| *Doryfera johannae* | dimorphic |  |  |  |  | 1 |  |  |  |  |  |  |  | 1 |  |  |  |
| *Doryfera ludovicae* | dimorphic |  |  |  |  | 1 |  |  |  |  |  |  |  | 1 |  |  |  |
| *Heliothryx barroti* | dimorphic |  |  |  |  | 1 |  |  |  |  |  |  |  | 1 |  |  |  |
| **Brilliants (Lesbiinae – Coeligenini)** |  |  |  |  |  |  |  |  |  |  |  |  |  |  |  |  |  |
| *Heliodoxa jacula* | dimorphic |  |  |  |  | 10 | 1 | 1 | 1 |  |  |  |  | 10 | 1 | 1 | 1 |
| **Mtn. Gems (Trochilinae – Lampornini)** |  |  |  |  |  |  |  |  |  |  |  |  |  |  |  |  |  |
| *Eugenes spectabilis* | dimorphic |  |  |  |  |  |  |  |  | 26 | 3 | 4 | 5 | 26 | 3 | 4 | 5 |
| *Heliomaster constantii* | dimorphic |  |  |  |  | 7 |  |  |  |  |  |  |  | 7 |  |  |  |
| *Lampornis calolaemus* | dimorphic |  |  |  |  | 1 |  |  |  | 28 | 4 | 6 | 7 | 29 | 4 | 6 | 7 |
| *Lampornis cinereicauda* | dimorphic |  |  |  |  |  |  |  |  | 5 | 2 | 1 | 2 | 5 | 2 | 1 | 2 |
| *Lampornis hemileucus* | dimorphic |  |  |  |  | 8 | 1 |  | 1 |  |  |  |  | 8 | 1 |  | 1 |
| *Panterpe insignis* | monomorphic |  |  |  |  |  |  |  |  | 42 | 4 | 3 | 5 | 42 | 4 | 3 | 5 |
| **Bees (Trochilinae – Mellisugini)** |  |  |  |  |  |  |  |  |  |  |  |  |  |  |  |  |  |
| *Selasphorus flammula* | dimorphic |  |  |  |  |  |  |  |  | 22 | 11 | 11 | 13 | 22 | 11 | 11 | 13 |
| **Emeralds (Trochilinae – Cynanthini)** |  |  |  |  |  |  |  |  |  |  |  |  |  |  |  |  |  |
| *Campylopterus hemileucurus* | dimorphic |  |  |  |  | 40 | 1 |  | 1 | 3 |  |  |  | 43 | 1 |  | 1 |
| *Cynanthus canivetii* | dimorphic | 4 |  |  |  | 3 | 1 |  | 1 |  |  |  |  | 7 | 1 |  | 1 |
| *Chlorostilbon lucidus* | dimorphic | 4 |  |  |  |  |  |  |  |  |  |  |  | 4 |  |  |  |
| *Klais guimeti* | dimorphic |  |  |  |  | 3 |  |  |  |  |  |  |  | 3 |  |  |  |
| *Stephanoxis loddigesii* | dimorphic | 1 |  |  |  |  |  |  |  |  |  |  |  | 1 |  |  |  |
| **Emeralds/Amazilias (Trochilinae – Trochilini)** |  |  |  |  |  |  |  |  |  |  |  |  |  |  |  |  |  |
| *Amazilia rutila* | dimorphic |  |  |  |  | 9 |  |  |  |  |  |  |  | 9 |  |  |  |
| *Amazilia tzacatl* | dimorphic | 46 |  | 3 | 3 | 4 |  | 1 | 1 |  |  |  |  | 50 |  | 4 | 4 |
| *Amazilis amazilia* | monomorphic | 1 |  |  |  |  |  |  |  |  |  |  |  | 1 |  |  |  |
| *Elliotomyia chionogaster* | monomorphic |  |  |  |  |  |  |  |  | 19 | 2 | 2 | 4 | 19 | 2 | 2 | 4 |
| *Eupherusa eximia* | dimorphic |  |  |  |  | 7 | 3 | 2 | 4 | 1 |  | 1 | 1 | 8 | 3 | 3 | 5 |
| *Eupherusa nigriventris* | dimorphic |  |  |  |  | 5 | 3 | 3 | 3 |  |  |  |  | 5 | 3 | 3 | 3 |
| *Hylocharis chrysura* | monomorphic | 7 |  |  |  |  |  |  |  |  |  |  |  | 7 |  |  |  |
| *Chalybura urochrysia* | dimorphic | 20 | 3 | 1 | 3 |  |  |  |  |  |  |  |  | 20 | 3 | 1 | 3 |
| *Chionomesa fimbriata* | monomorphic | 1 |  |  |  |  |  |  |  |  |  |  |  | 1 |  |  |  |
| *Chionomesa lactea* | monomorphic |  |  |  |  | 2 |  |  |  |  |  |  |  | 2 |  |  |  |
| *Chlorestes candida* | monomorphic | 13 | 1 | 2 | 2 |  |  |  |  |  |  |  |  | 13 | 1 | 2 | 2 |
| *Chlorestes eliciae* | dimorphic | 2 |  |  |  |  |  |  |  |  |  |  |  | 2 |  |  |  |
| *Microchera cupreiceps* | dimorphic |  |  |  |  | 1 | 1 | 1 | 1 |  |  |  |  | 1 | 1 | 1 | 1 |
| *Microchera chionura* | dimorphic |  |  |  |  | 1 |  |  |  |  |  |  |  | 1 |  |  |  |
| *Phaeochroa cuvierii* | monomorphic |  |  |  |  | 3 |  |  |  |  |  |  |  | 3 |  |  |  |
| *Polyerata amabilis* | dimorphic | 4 |  |  |  |  |  |  |  |  |  |  |  | 4 |  |  |  |
| *Saucerottia edward* | monomorphic |  |  |  |  | 1 |  |  |  |  |  |  |  | 1 |  |  |  |
| *Saucerottia hoffmanni* | dimorphic |  |  |  |  | 4 |  |  |  |  |  |  |  | 4 |  |  |  |
| *Thalurania colombica* | dimorphic | 4 |  |  |  | 1 |  |  |  |  |  |  |  | 5 |  |  |  |
| *Thalurania furcata* | dimorphic | 1 |  |  |  |  |  |  |  |  |  |  |  | 1 |  |  |  |
| **Total number of birds** |  | 252 | 17 | 12 | 24 | 176 | 16 | 11 | 19 | 151 | 26 | 28 | 37 | 579 | 59 | 51 | 80 |
| **Number of species** |  | 22 | 4 | 6 | 6 | 29 | 11 | 7 | 12 | 9 | 5 | 6 | 6 | 49 | 19 | 18 | 22 |
| **Prevalence (%)** |  |  | 6.7 | 4.8 | **9.5** |  | 9.1 | 6.3 | **10.8** |  | 17.2 | 18.5 | **24.5** |  | 10.2 | 8.8 | **13.8** |
|  |  |  |  |  |  |  |  |  |  |  |  |  |  |  |  |  |  |
| Data for species with ≥ 10 individuals examined |  |  |  |  |  |  |  |  |  |  |  |  |  |  |  |  |  |
| **Total number of birds** |  | 212 | 17 | 11 | 23 | 108 | 4 | 3 | 6 | 140 | 24 | 26 | 34 | 460 | 45 | 40 | 63 |
| **Number of species** |  | 8 | 4 | 5 | 5 | 8 | 4 | 3 | 5 | 6 | 5 | 5 | 5 | 16 | 12 | 12 | 13 |
| **Prevalence (%)** |  |  | 8.0 | 5.2 | **10.8** |  | 3.7 | 2.8 | **5.6** |  | 17.1 | 18.6 | **24.3** |  | 9.8 | 8.7 | **13.7** |

**Additional file 1: Table S6.** Prevalence of chewing lice on young (young-of-the-year) and adult hummingbirds (only species for which we were able to determine the age of at least some individuals are shown).

P = number of birds parasitized with lice; E = number of birds examined

|  | **Total** | | **Undetermined** | | **Young** | | **Adult** | | **Prevalence (%)** | |
| --- | --- | --- | --- | --- | --- | --- | --- | --- | --- | --- |
|  | **P** | **E** | **P** | **E** | **P** | **E** | **P** | **E** | **young** | **adult** |
| *Amazilia tzacatl* | 4 | 50 | 3 | 37 | 1 | 7 | 0 | 6 | 14 | 0 |
| *Campylopterus hemileucurus* | 1 | 43 | 1 | 36 | 0 | 7 | 0 | 0 | 0 | – |
| *Cynanthus canivetii* | 1 | 7 |  | 6 | 1 | 1 | 0 | 0 | 100 | – |
| *Eupherusa eximia* | 5 | 8 | 3 | 5 | 2 | 3 | 0 | 0 | 67 | – |
| *Eutoxeres aquila* | 0 | 13 | 0 | 2 | 0 | 0 | 0 | 11 | – | – |
| *Glaucis aeneus* | 0 | 10 | 0 | 0 | 0 | 2 | 0 | 8 | 0 | 0 |
| *Chalybura urochrysia* | 3 | 20 | 0 | 5 | 0 | 0 | 3 | 15 | – | 20 |
| *Microchera cupreiceps* | 1 | 1 |  |  | 1 | 1 | 0 | 0 | 100 | - |
| *Phaethornis longirostris* | 11 | 74 | 11 | 39 | 0 | 0 | 0 | 35 | – | 0 |
| *Phaethornis striigularis* | 2 | 15 | 2 | 6 | 0 | 0 | 0 | 9 | – | 0 |
| *Polyerata amabilis* | 0 | 4 | 0 | 1 | 0 | 2 | 0 | 1 | 0 | 0 |
| *Selasphorus flammula* | 14 | 23 | 12 | 21 | 2 | 2 | 0 | 0 | 100 | – |
| *Thalurania colombica* | 0 | 5 | 0 | 3 | 0 | 0 | 0 | 2 | – | 0 |
| *Threnetes ruckeri* | 5 | 30 | 2 | 7 | 0 | 0 | 3 | 23 | – | 13 |
| Total | 47 | 303 | 34 | 168 | 7 | 25 | 6 | 110 | **28** | **5.5** |
| Data for species with ≥ 10 individuals determined |  |  |  |  | 1 | 9 | 6 | 98 | **11** | **6.1** |

**Additional file 1: Table S6.** Prevalence, mean intensity, mean abundance, sex ratio and age ratio of four genera of chewing lice found on hummingbirds. P = number of birds parasitized; E = number of birds examined

| **Lice genus** | **Hosts** | **P** | **E** | **Prevalence (%)** | **Females** | **Males** | **Nymphs** | **Total** | **Intensity** | **Abundance** | **% of male lice** | **% of adult lice** |
| --- | --- | --- | --- | --- | --- | --- | --- | --- | --- | --- | --- | --- |
| *Trochiloecetes* | total | 44 | 579 | 7.6 | 101 | 50 | 67 | 218 | 5.0 | 0.4 | 33.1 | 69.3 |
| *Trochiliphagus* | total | 14 | 579 | 2.4 | 14 | 10 | 17 | 41 | 2.9 | 0.1 | 41.7 | 58.5 |
| *Myrsidea* | total | 1 | 579 | 0.2 | 0 | 1 | 6 | 7 | 7.0 | 0.01 | 100 | 14.3 |
| *Leremenopon* | total | 1 | 579 | 0.2 | 1 | 0 | 0 | 1 | 1.0 | 0.002 | 0 | 100 |
| Total | total | 59* | 579 | 10.2 | 116 | 61 | 90 | 267 | 4.5 | 0.5 | 34.5 | 66.3 |
|  |  |  |  |  |  |  |  |  |  |  |  |  |
| *Trochiloecetes* | females of hosts | 5 | 95 | 5.3 | 21 | 11 | 13 | 45 | 9.0 | 0.5 | 34.4 | 71.1 |
| *Trochiloecetes* | males of hosts | 6 | 71 | 8.5 | 3 | 2 | 8 | 13 | 2.2 | 0.2 | 40.0 | 38.5 |
| *Trochiloecetes* | dimorphic | 26 | 276 | 9.4 | 48 | 24 | 40 | 112 | 4.3 | 0.4 | 33.3 | 64.3 |
| *Trochiloecetes* | monomorphic | 18 | 303 | 5.9 | 53 | 26 | 27 | 106 | 5.9 | 0.3 | 32.9 | 74.5 |
| *Trochiloecetes* | lowlands | 14 | 252 | 5.6 | 33 | 4 | 27 | 64 | 4.6 | 0.3 | 10.8 | 57.8 |
| *Trochiloecetes* | highlands | 9 | 176 | 5.1 | 29 | 18 | 23 | 70 | 7.8 | 0.4 | 38.3 | 67.1 |
| *Trochiloecetes* | mountains | 21 | 151 | 13.9 | 39 | 28 | 17 | 84 | 4.0 | 0.6 | 41.8 | 79.8 |
|  |  |  |  |  |  |  |  |  |  |  |  |  |
| *Trochiliphagus* | females of hosts | 2 | 95 | 2.1 | 1 | 0 | 1 | 2 | 1.0 | 0.02 | 0 | 50.0 |
| *Trochiliphagus* | males of hosts | 5 | 71 | 7.0 | 8 | 2 | 7 | 17 | 3.4 | 0.2 | 20.0 | 58.8 |
| *Trochiliphagus* | dimorphic | 11 | 276 | 4.0 | 11 | 7 | 12 | 30 | 2.7 | 0.1 | 38.9 | 60.0 |
| *Trochiliphagus* | monomorphic | 3 | 303 | 1.0 | 3 | 3 | 5 | 11 | 3.7 | 0.04 | 50.0 | 54.5 |
| *Trochiliphagus* | lowlands | 1 | 252 | 0.4 | 0 | 3 | 5 | 8 | 8.0 | 0.03 | 100.0 | 37.5 |
| *Trochiliphagus* | highlands | 7 | 176 | 4.0 | 4 | 1 | 5 | 10 | 1.4 | 0.1 | 20.0 | 50.0 |
| *Trochiliphagus* | mountains | 6 | 151 | 4.0 | 10 | 6 | 7 | 23 | 3.8 | 0.2 | 37.5 | 69.6 |

* = one bird was parasitized by lice of two genera

**Additional file 1: Table S7.** Comparison of prevalence found at present study with those reported by Clayton et al. (1992) and Oniki-Willis et al. (2023). P = number of birds parasitized; E = number of birds examined.

|  | ***Trochiloecetes*** | | | | | | ***Trochiliphagus*** | | | | | |
| --- | --- | --- | --- | --- | --- | --- | --- | --- | --- | --- | --- | --- |
|  | **This study** | | **Oniki-Willis et al. (2023)** | | **Clayton et al. (1992)** | | **This study** | | **Oniki-Willis et al. (2023)** | | **Clayton et al. (1992)** | |
| Host | P/E | prev.(%) | P/E | prev.(%) | P/E | prev.(%) | P/E | prev.(%) | P/E | prev.(%) | P/E | prev.(%) |
| *Amazilia tzacatl* | 4*/50 | 8.0 | 90/996 | 9.0 |  |  | 4*/50 | 8.0 | 141/996 | 14.2 |  |  |
| *Anthracothorax prevostii* | 1*/3 | 33.3 | 2/191 | 1.0 |  |  | 1*/3 | 33.3 | 15/191 | 7.9 |  |  |
| *Campylopterus hemileucurus* | 1/43 | 2.3 | 13/276 | 4.7 |  |  | 0/43 | 0 | 0/276 | 0 |  |  |
| *Chalybura urochrysia* | 2/20 | 10.0 | 30/166 | 18.1 |  |  | 0/20 | 0 | 25/166 | 15.1 |  |  |
| *Chlorestes candida* | 0/13 | 0 | 9/203 | 4.4 |  |  | 1/13 | 7.7 | 15/203 | 7.4 |  |  |
| *Cynanthus canivetii* | 0/7 | 0 | 13/204 | 6.4 |  |  | 1/7 | 14.3 | 19/204 | 9.3 |  |  |
| *Doryfera ludovicae* | 0/1 | 0 | 0/206 | 0 |  |  | 0/1 | 0 | 0/206 | 0 | 1/19 | 5 |
| *Elliotomyia chionogaster* | 1/19 | 5.3 | 2/122 | 1.6 |  |  | 1/19 | 5.3 | 11/122 | 9.0 |  |  |
| *Eugenes spectabilis* | 1/26 | 3.8 | 3/34 | 8.8 |  |  | 2/26 | 7.7 | 10/34 | 29.4 |  |  |
| *Eupherusa eximia* | 2/8 | 25.0 | 51/214 | 23.8 |  |  | 1/8 | 12.5 | 10/214 | 4.7 |  |  |
| *Eupherusa nigriventris* | 1/5 | 20.0 | 9/41 | 22.0 |  |  | 2/5 | 40.0 | 6/41 | 14.6 |  |  |
| *Heliodoxa jacula* | 0/10 | 0 | 12/139 | 8.6 |  |  | 1/10 | 10.0 | 10/139 | 7.2 |  |  |
| *Klais guimeti* | 0/3 | 0 | 15/173 | 8.7 |  |  | 0/3 | 0 | 14/173 | 8.1 | 1/2 | 50 |
| *Lampornis calolaemus* | 4/29 | 13.8 | 1/37 | 2.7 |  |  | 0/29 | 0 | 1/37 | 2.7 |  |  |
| *Lampornis cinereicauda* | 2/5 | 40.0 | 13/128 | 10.2 |  |  | 0/5 | 0 | 2/128 | 1.6 |  |  |
| *Lampornis hemileucus* | 0/8 | 0 | 6/38 | 15.8 |  |  | 1/8 | 12.5 | 2/38 | 5.3 |  |  |
| *Microchera cupreiceps* | 1/1 | 100 | 15/95 | 15.8 |  |  | 0/1 | 0 | 8/95 | 8.4 |  |  |
| *Panterpe insignis* | 4/42 | 9.5 | 25/233 | 10.7 |  |  | 0/42 | 0 | 2/233 | 0.9 |  |  |
| *Phaethornis atrimentalis* | 2/4 | 50.0 | 3/20 | 15.0 |  |  | 0/4 | 0 | 2/20 | 10.0 |  |  |
| *Phaethornis guy* | 0/43 | 0 | 8/259 | 3.1 | 3/17 | 18 | 0/43 | 0 | 1/259 | 0.4 |  |  |
| *Phaethornis longirostris* | 9/75 | 12.0 | 2/46 | 4.3 |  |  | 0/75 | 0 | 6/46 | 13.0 |  |  |
| *Phaethornis striigularis* | 0/15 | 0 | 1/37 | 2.7 |  |  | 1/15 | 6.7 | 5/37 | 13.5 |  |  |
| *Selasphorus flammula* | 9/22 | 40.9 | 37/174 | 21.3 |  |  | 3/22 | 13.6 | 50/174 | 28.7 |  |  |
| *Threnetes leucurus* | 1/3 | 33.3 | 14/129 | 10.9 |  |  | 0/3 | 0 | 2/129 | 1.6 | 1/7 | 29 |
| *Threnetes ruckeri* | 4/30 | 13.3 | 26/210 | 12.4 | 1/7 | 29 | 0/30 | 0 | 13/210 | 6.2 |  |  |
| **Total** | **44/579** | **7.6** | **7106/**  **74760** | **9.5** | **14/225** | **6.2** | **14/579** | **2.4** | **3868/**  **74760** | **5.2** | **12/225** | **5.3** |

*=only louse eggs were found on these hosts

**Additional file 1: Table S8.** D-correlation matrix

Following variables are included A) hummingbirds’ characteristics: 1 Mean_mass – mean body mass, 2 Mass_dim – sexual size dimorphism, 3 Colour_dim – sexual dichromatism, 4 A migrant – migrant behavior, 5 B migrant – type of migration, 6 Behav_dom – behavioral dominance; B) characteristics of geographic areas: 7 MeanAlt – mean elevation, 8 Temp_mean – mean temperature, 9 Temp_Pred – predictability of temperature, 10 Prec_mean – mean precipitation, 11 Prec_Pred – predictability of precipitation; C) infestation indices: 12 AllLicePrev – prevalence of lice, 13 AllLiceEggP – prevalence of lice and louse eggs or nits, 14 AllMeanAbun – mean abundance of lice, 15 Toe_Prev – prevalence of *Trochiloecetes* lice, 16 ToeMeanAbun – mean abundance of *Trochiloecetes* lice, 17 Tph_Prev – prevalence of *Trochiliphagus* lice, 18 TphMeanAbun – mean abundance of *Trochiliphagus* lice.

|  | 1 | 2 | 3 | 4 | 5 | 6 | 7 | 8 | 9 | 10 | 11 | 12 | 13 | 14 | 15 | 16 | 17 | 18 |
| --- | --- | --- | --- | --- | --- | --- | --- | --- | --- | --- | --- | --- | --- | --- | --- | --- | --- | --- |
| 1 Mean_mass | 1.0000 | .1279 | -.0191 | .0785 | .1006 | .0437 | -.0775 | -.1412 | -.0574 | -.1567 | -.1515 | -.2861 | -.2628 | -.0554 | -.2658 | .0287 | -.1866 | -.2808 |
| 2 Mass_dim | .1279 | 1.0000 | .0144 | .0663 | .0725 | .0022 | .2217 | -.1892 | .1175 | .0986 | -.0916 | .0909 | .0510 | .0460 | .0648 | .0561 | .0606 | -.0364 |
| 3 Colour_dim | -.0191 | .0144 | 1.0000 | -.0254 | -.0485 | .0740 | -.0572 | -.0217 | .0361 | .0265 | -.0347 | -.0737 | -.0740 | -.0466 | -.0702 | -.0367 | -.0657 | -.0455 |
| 4 A migrant | .0785 | .0663 | -.0254 | 1.0000 | .9621 | .1272 | .5219 | .2427 | .0974 | .1484 | .1291 | .3015 | .3093 | .1344 | .2344 | .0776 | .2704 | .2227 |
| 5 B migrant | .1006 | .0725 | -.0485 | .9621 | 1.0000 | .1321 | .5167 | .2390 | .1667 | .2101 | .1454 | .3149 | .3234 | .1393 | .2464 | .0792 | .2842 | .2340 |
| 6 Behav_dom | .0437 | .0022 | .0740 | .1272 | .1321 | 1.0000 | .1145 | -.0913 | -.0428 | .0262 | .0002 | -.0734 | -.0900 | -.0537 | -.0742 | -.0369 | -.0171 | -.0669 |
| 7 MeanAlt | -.0775 | .2217 | -.0572 | .5219 | .5167 | .1145 | 1.0000 | -.5451 | .1441 | .1571 | -.5206 | .3605 | .3900 | .3089 | .3064 | .1975 | .3294 | .4202 |
| 8 Temp_mean | -.1412 | -.1892 | -.0217 | .2427 | .2390 | -.0913 | -.5451 | 1.0000 | .3032 | .2868 | .5777 | -.0692 | -.0927 | -.0289 | .0070 | .0519 | -.2811 | -.2781 |
| 9 Temp_Pred | -.0574 | .1175 | .0361 | .0974 | .1667 | -.0428 | .1441 | .3032 | 1.0000 | .8249 | .3445 | .3883 | .3610 | .2749 | .3643 | .2250 | .1919 | .2155 |
| 10 Prec_mean | -.1567 | .0986 | .0265 | .1484 | .2101 | .0262 | .1571 | .2868 | .8249 | 1.0000 | .2771 | .4720 | .4293 | .2996 | .4360 | .2188 | .2524 | .3145 |
| 11 Prec_Pred | -.1515 | -.0916 | -.0347 | .1291 | .1454 | .0002 | -.5206 | .5777 | .3445 | .2771 | 1.0000 | -.1630 | -.2190 | .0097 | -.0970 | .0816 | -.2667 | -.2288 |
| 12 AllLicePrev | -.2861 | .0909 | -.0737 | .3015 | .3149 | -.0734 | .3605 | -.0692 | .3883 | .4720 | -.1630 | 1.0000 | .9586 | .6405 | .9478 | .5241 | .4699 | .4938 |
| 13 AllLiceEggP | -.2628 | .0510 | -.0740 | .3093 | .3234 | -.0900 | .3900 | -.0927 | .3610 | .4293 | -.2190 | .9586 | 1.0000 | .6292 | .8990 | .5081 | .4841 | .5105 |
| 14 AllMeanAbun | -.0554 | .0460 | -.0466 | .1344 | .1393 | -.0537 | .3089 | -.0289 | .2749 | .2996 | .0097 | .6405 | .6292 | 1.0000 | .6147 | .9525 | .2809 | .3737 |
| 15 Toe_Prev | -.2658 | .0648 | -.0702 | .2344 | .2464 | -.0742 | .3064 | .0070 | .3643 | .4360 | -.0970 | .9478 | .8990 | .6147 | 1.0000 | .5651 | .1719 | .2844 |
| 16 ToeMeanAbun | .0287 | .0561 | -.0367 | .0776 | .0792 | -.0369 | .1975 | .0519 | .2250 | .2188 | .0816 | .5241 | .5081 | .9525 | .5651 | 1.0000 | .0356 | .0777 |
| 17 Tph_Prev | -.1866 | .0606 | -.0657 | .2704 | .2842 | -.0171 | .3294 | -.2811 | .1919 | .2524 | -.2667 | .4699 | .4841 | .2809 | .1719 | .0356 | 1.0000 | .8246 |
| 18 TphMeanAbun | -.2808 | -.0364 | -.0455 | .2227 | .2340 | -.0669 | .4202 | -.2781 | .2155 | .3145 | -.2288 | .4938 | .5105 | .3737 | .2844 | .0777 | .8246 | 1.0000 |
